# Model-Based Planning and Delivery of Mass Vaccination Campaigns against Infectious Disease: Application to the COVID-19 Pandemic in the UK

**DOI:** 10.3390/vaccines9121460

**Published:** 2021-12-10

**Authors:** Dauda Ibrahim, Zoltán Kis, Kyungjae Tak, Maria M. Papathanasiou, Cleo Kontoravdi, Benoît Chachuat, Nilay Shah

**Affiliations:** 1 The Sargent Centre for Process Systems Engineering, Department of Chemical Engineering, Imperial College London, London SW7 2AZ, UK; z.kis10@imperial.ac.uk (Z.K.); k.tak@imperial.ac.uk (K.T.); maria.papathanasiou11@imperial.ac.uk (M.M.P.); cleo.kontoravdi98@imperial.ac.uk (C.K.); b.chachuat@imperial.ac.uk (B.C.); n.shah@imperial.ac.uk (N.S.); 2 Department of Chemical and Biological Engineering, The University of Sheffield, Sheffield S1 3JD, UK

**Keywords:** SARS-CoV-2 vaccines, vaccination campaign, vaccine availability, demand stratification, economic analysis, mathematical programming

## Abstract

Vaccination plays a key role in reducing morbidity and mortality caused by infectious diseases, including the recent COVID-19 pandemic. However, a comprehensive approach that allows the planning of vaccination campaigns and the estimation of the resources required to deliver and administer COVID-19 vaccines is lacking. This work implements a new framework that supports the planning and delivery of vaccination campaigns. Firstly, the framework segments and priorities target populations, then estimates vaccination timeframe and workforce requirements, and lastly predicts logistics costs and facilitates the distribution of vaccines from manufacturing plants to vaccination centres. The outcomes from this study reveal the necessary resources required and their associated costs ahead of a vaccination campaign. Analysis of results shows that by integrating demand stratification, administration, and the supply chain, the synergy amongst these activities can be exploited to allow planning and cost-effective delivery of a vaccination campaign against COVID-19 and demonstrates how to sustain high rates of vaccination in a resource-efficient fashion.

## 1. Introduction

Severe acute respiratory syndrome coronavirus 2 (SARS-CoV-2), a virus that causes the coronavirus 2019 (COVID-19) disease, was first identified in December 2019 [1,2]. Due to the rapid spread and highly contagious nature of this new coronavirus, the World Health Organisation (WHO) declared COVID-19 a pandemic on 11 March 2020 [1,2,3,4], prompting governments around the world to take swift measures aimed at mitigating the economic, health, and social impacts of the disease. Supported by governments and non-governmental organisations [5,6,7,8], pharmaceutical companies and academic institutions have deployed a range of platform technologies to produce COVID-19 vaccines in record time, including: (i) viral vectors such as AZD1222 (ChAdOx1 nCoV2) developed by University of Oxford and AstraZeneca [9,10], Ad26.COV2.S developed by Johnson and Johnson [11,12,13], and Sputnik V (rAd26 and rAd5) developed by Gamaleya Research Institute [14,15,16]; (ii) nucleic-acid-based vaccines such as BNT162b2 developed by Pfizer and BioNTech [17,18,19], mRNA-1273 developed by Moderna [20,21,22], and CVnCoV developed by CureVac [23]; (iii) inactivated pathogens such as BBIBP-CorV developed by Sinopharm [24]; (iv) protein subunits such as NVX-CoV2373 developed by Novavax [25,26].

Despite the successes recorded in the development of vaccines against SARS-CoV-2, many challenges remain in the deployment and administration of COVID-19 vaccines in both low- and middle-income countries (LMICs) and high-income countries (HICs) [27,28,29,30,31,32]. Key challenges include (i) the need to vaccinate the world population rapidly to achieve herd immunity; (ii) scaling-up vaccine manufacturing capacity and the vaccine supply chain to meet the global demand for SARS-CoV-2 vaccines; (iii) developing supply chains capable of handling viral vectors, nucleic-acid-based vaccines, inactivated pathogens, and protein subunits; and (iv) building resilience in vaccine supply chains that can simultaneously handle routine immunisation and mass vaccination campaigns against infectious diseases such as COVID-19. Another activity to be conducted before the implementation of a mass vaccination campaign is the assessment of the relative risks and benefits of the intervention, taking into account the following: (i) the potential impact of the vaccine-preventable disease or high-impact disease outbreak using key epidemiological criteria; (ii) the potential benefits of a mass vaccination campaign and the country’s capacity to implement it safely and effectively; (iii) the potential risk of increased COVID-19 transmission associated with the mass vaccination campaign; (iv) determination of the most appropriate actions considering the epidemiological situation related to COVID-19; and (v) implementation of best practice if a decision is made to proceed with a mass vaccination campaign [33]. The detailed standard criteria recommended by the WHO on how to assess the risks and benefits of conducting a mass vaccination campaign can be found elsewhere [33].

The use of mathematical models to address these challenges and to inform decision-making related to public health has intensified in recent years. Such models have been developed and applied to facilitate understanding and to analyse the spread of infectious diseases [34,35,36,37,38,39], vaccine manufacture processes [40,41,42,43,44,45], and vaccine supply chains [46,47,48,49]. Vaccine supply chains in particular are complex networks that store and transport vaccines under controlled temperature from manufacturing plants to vaccination centres. There are two categories of models typically employed to describe them: simulation-based models and optimisation-based models. The former evaluate one vaccine distribution scenario at a time and can be used in designing new supply chains or re-designing existing ones to improve a specific performance indicator such as cost or vaccine availability [49,50,51]. In contrast, optimisation-based models support the use of optimisation algorithms to identify the best candidate supply chain among multiple alternatives. The advantages of optimisation-based models include the optimal selection of locations for entities such as manufacturing plants, warehouses, and regional stores; the identification of transport routes that minimise delivery time and transportation costs; the determination of the target capacities of manufacturing plants, fill-finish plants, warehouses, and administration points; and supporting the evaluation of several key performance indicators, such as the total logistic costs, vaccine availability, lead time, and storage capacity utilisation.

Optimisation-based models have been used to design and plan sustainable vaccine supply chains [52]; to investigate the impact of uncertain parameters such as vaccine demand on supply chain performance [53]; to plan the distribution for an expanded programme of immunisation vaccines in LMICs [54]; to assess the performance of vaccine supply chains in LMICs, considering the distribution of vaccines produced using new manufacturing technologies such as RNA vaccines, outer membrane vesicle vaccines with genetically customisable membrane antigens (customOMV), virus-like particle vaccines with genetically configurable epitopes (customVLP), and humanised yeast-produced vaccines [55]; and to plan the distribution of COVID-19 vaccines during the ongoing pandemic [56]. These previous studies have focused on the design and analyses of vaccine supply chains only, without considering vaccine demand stratification and the planning of vaccine administration. Ahead of a mass vaccination campaign, demand stratification may be applied to segment and prioritise a target population in order to maximise the effectiveness of vaccination, while the planning of vaccine administration involves estimation of the vaccination timeframe and regional workforce requirements in order to avoid failure to meet vaccination targets and to reduce vaccine wastage and the number of missed vaccination opportunities at clinics, hospitals, pharmacies, and vaccination centres. A lack of proper planning could compromise the effectiveness of vaccination, leading to a surge in the number of cases and increased death and hospitalisation rates.

Additionally, models presented in previous works do not support (i) the handling of passive cold chain devices such as thermal shippers, (ii) quality control testing at warehouses, or (iii) vaccine supply from in-country and overseas manufacturing and fill-finish facilities. These are essential components of a typical vaccine supply chain that should be taken into account in order to maintain integrity of vaccines throughout the cold chain and to ensure that safe, potent, and efficacious vaccines are delivered to the right places and at the right time, especially during a pandemic.

This paper presents an optimisation-based model for vaccine supply chains, which is embedded within a systematic framework combining vaccine demand stratification, vaccine administration, and vaccine supply and delivery. A distinctive feature of our supply chain model is that it considers the storage and transportation of vaccines at both refrigerated and ultra-low temperatures, which is an essential requirement for nucleic-acid-based vaccines (e.g., mRNA vaccines), as well as the handling of passive cold chain devices such as vaccine thermal shippers during a vaccination campaign. The model also includes quality control test capabilities at warehouses, such that each batch of vaccine is tested before distribution to vaccination centres and can handle various types of vaccines, such as viral vectors, nucleic-acid-based vaccines, inactivated pathogens, protein subunits, and conjugate vaccines. We use real-world data to demonstrate the applicability of the proposed framework, focusing on determining the resources required, from a supply chain and vaccine administration perspective, to vaccinate up to 500,000 patients per day against COVID-19 within the UK.

The remainder of this article is organised as follows. Section 2 introduces the case study on COVID-19 vaccination in the UK, which is used to demonstrate the applicability of the proposed framework. Section 3 outlines the proposed framework and discusses, in detail, all components of the framework. Section 4 concludes the paper and presents future work directions.

## 2. Methods

### 2.1. Modelling Framework

**Figure 1:**
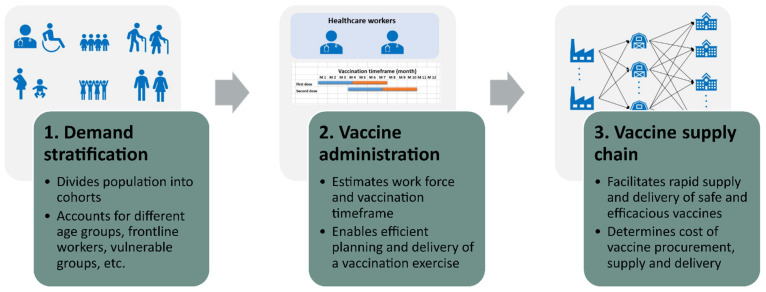
Modelling framework for the supply, distribution, and administration of COVID-19 vaccine, showing the direction of information.

Figure 1 shows the proposed systematic framework that supports vaccine supply and administration. The framework comprises three distinct steps that model vaccine demand stratification, vaccine administration, and vaccine supply and delivery.

Step 1. Given the total population of a geographical area and cohort size, the demand stratification model estimates the number of individuals belonging to each cohort in all regions within a geographical area; see Supplementary 4 for further information. This information is critical since it enables a specific segment of a population to be targeted during a vaccination campaign against infectious diseases;

Step 2. The vaccine administration model computes the duration needed to vaccinate each cohort determined in step 1, the vaccination schedule, and the total number of healthcare personnel needed to implement the vaccination exercise see Supplementary 4 for further information. In addition to stratified demand for all regions, other inputs to this model include staff working hours per day, vaccine administration rate, and patients vaccinated per day;

Step 3. The vaccine supply chain model makes use of information determined from the demand stratification and vaccine administration in steps 1 and 2 to predict logistics operations and costs and to optimise the supply, delivery, and administration of various vaccine candidates; see Section 2.2 below and Supplementary 3 for further details.

Demand stratification, vaccine administration, and vaccine supply and distribution are inter-dependent. For example, modifying the target population or cohort size will affect workforce requirements and the vaccination timeframe, which in turn will affect vaccine supply, and ultimately logistics cost. Using the proposed framework, the synergies amongst these activities can be exploited to minimise logistics cost and to allow efficient planning and delivery of a vaccination campaign. This cannot be achieved using previously published methodologies.

### 2.2. Vaccine Supply Chain Modelling

**Figure 2:**
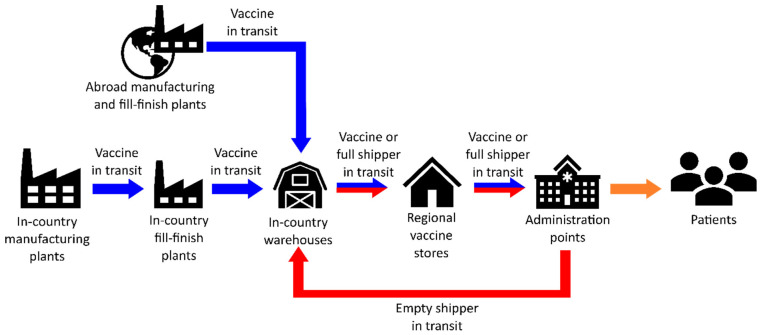
Schematic of the proposed vaccine supply chain comprising internal and external manufacturing and fill-finish plants, in-country warehouses, regional vaccine stores, and administration points (GP surgeries, hospitals, pharmacies, vaccination centres, etc.). Vaccines flow from manufacturing facilities to administration points via transport devices (plane, refrigerated van, refrigerated truck, etc.). The flows of vaccines and thermal shippers are indicated by the blue and red lines, respectively. The orange line indicates the flow of vaccines out of the supply chain for administration to target vaccinees.

The cornerstone of the modelling framework is a mixed-integer linear programming (MILP) model describing a multi-echelon supply chain (see Figure 2) for the distribution and delivery of vaccine candidates from plants to vaccination centres.

The supply chain comprises five echelons: manufacturing, fill-finish, warehouse, regional store, and administration point. Vaccine manufacturing and fill-finish can be carried either in-country, abroad, or both. Vaccine drug substances flow from manufacturing plants to fill-finish plants, where they are filled into sterile glass vials or bags and packaged into cartons. The packaged vaccines are then shipped to warehouses, followed by regional stores. Administration points (GP surgeries, hospitals, pharmacies, vaccination centres, etc.) receive vaccines from regional stores based on demand, which is determined by the number of appointments registered. Throughout the supply chain, in-country distributions are fulfilled using refrigerated vans or refrigerated trucks, while vaccine imports are carried out using air freight.

The mathematical formulation of the supply chain model developed in this work is presented in Supplementary 3. Essential input parameters to the model include:

1. Vaccine demand profile;
2. Vaccination timeframe;
3. Supply chain superstructure;
4. Minimum and maximum inventories (manufacturing and fill-finish, warehouses, regional stores, and administration points);
5. Minimum and maximum capacities of manufacturing plants, fill-finish plants, and import rate;
6. Minimum and maximum capacities of transportation modes, operating costs and capital cost factors (manufacturing and fill-finish, warehouses, regional stores, and administration points);
7. Travel distances and times.

Using these inputs, the model calculates the following outputs:

1. Optimal supply chain structure;
2. Transport mode per route;
3. Backlog in each time period;
4. Vaccine availability and vaccine wastage at administration points;
5. Vaccine supplied to administration points per time period;
6. Vaccine import rate and production rates in manufacturing and fill-finish plants;
7. Capacity of quality control facilities;
8. Capital costs, operating costs, and total annualised cost of supply chain facilities;
9. Total transportation costs and transport costs per route;
10. Inventories of vaccines in manufacturing facilities, fill-finish facilities, warehouses, regional stores, and administration points;
11. Inventories of vaccine thermal shippers (full and empty) in warehouses, regional stores, and administration points (needed only for vaccines stored and transported at ultra-low temperatures).

In addition to optimising the flow of vaccines from manufacturing plants to administration points, the model can also be used to optimise the supply chain structure and operation, as well as to determine the best vaccination plan over a given time horizon. The optimisation of the vaccine supply chain enables improvement of key performance indicators such as the backlog, total investment cost, vaccine availability, lead time, cost per fully immunised patient, and logistic cost per dose. The type of objective function to be employed depends on the intended purpose of the vaccine supply chain or vaccination programme.

This work focuses on minimisation of the total annualised logistics cost and minimisation of the backlog, owing to their relevance not only in vaccine supply and distribution but also in other healthcare supply chains. Total annualised logistics cost is the sum of installed capital costs, operating costs, and transportation costs, while the backlog is the sum of unfulfilled or late vaccination or failed appointments over the entire vaccination period. Although this work aims to investigate the impact of vaccine supply chain operational and structural decisions on total annualised logistics costs and the backlog, the proposed model can also estimate other key performance indicators, including the logistics cost per fully immunised patient, logistics cost per dose, capacity utilisation, vaccine availability, vaccine wastage, and demand satisfaction.

### 2.3. Data Sources and Assumptions

#### 2.3.1. Demand Stratification

The essential information required to carry out a demand stratification is the geographical population and target group or cohort size information. Herein, we make use of data provided by the UK’s Office for National Statistics [57]. These data include five age cohort populations in 12 regions, namely the North East, North West, Yorkshire and the Humber, East Midlands, West Midlands, East, London, South East, South West, Wales, Northern Ireland, and Scotland. The population data are from 2020 population estimates, while stratification is conducted in agreement with the cohort size recommended by the Joint Committee on Vaccination and Immunisation (JCVI) [58].

#### 2.3.2. Vaccine Administration

In addition to the demand stratification, this step requires data on staff working hours per day, vaccine administration rate, and patients vaccinated per day (UK target). Herein, we assume that staff should work at least six hours a day and spend ten min vaccinating each patient, including screening and consent activities [59,60,61]. Following the UK government vaccination campaign ambitions, we assume a daily vaccination rate of 500,000 patients per day across the UK [62,63,64]. According to the British Medical Association (BMA), the cost of administrating a vaccine at GP surgeries, hospitals, pharmacies, and vaccination centres is equivalent to $16.80 (£12.58) per dose, while vaccines administered at care homes incur an extra $13.36 (£10) per dose [65]. We assume that care home residents and residential care workers are vaccinated within the premises of care homes.

#### 2.3.3. Vaccine Supply Chain

This is the most computationally demanding step of the framework and it requires an extensive input dataset. Since the model is driven by demand at vaccination centres or care homes, information related to the demand profile needs to be specified. The demand profile at each vaccination centre across the UK is fixed by the number of weekly appointments, which can be calculated using stratified demand or the number of individuals arriving at clinics in order to be inoculated with a jab. In our initial studies, we assume 100% coverage in all geographical regions in the UK. A vaccination schedule can be defined from the estimated time required to vaccinate each cohort in addition to the intervals between doses.

**Figure 3:**
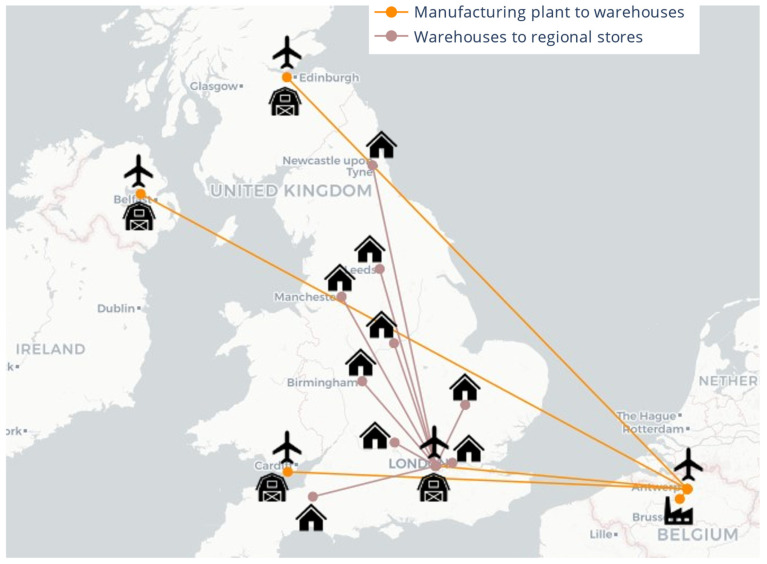
Supply chain for the distribution and delivery of the BNT162b2 SARS-CoV-2 vaccine candidate across the UK. No in-country vaccine manufacture or fill-finish facilities.

The supply chain structure is configured to mimic the existing vaccine supply and distribution network in the UK and is represented in Figure 3. The network consists of manufacturing and fill-finish plants co-located in Puur, Belgium; four warehouses located in London, Cardiff, Edinburgh, and Belfast; and 12 regional stores located in Cardiff, Edinburgh, Belfast, and the nine regions in England (North East, North West, Yorkshire and the Humber, East Midlands, West Midlands, East of England, London, South East, South West). Within the UK, the government aims to established several vaccination centres within a ten-mile radius of the surrounding populace [66], leading to spatially distributed vaccine administration points. To reduce the computational load during simulation and optimisation studies, we group all vaccination centres in each region.

The working assumption is that sufficient vaccines can be supplied from plants on weekly basis, meaning there is no limitation on vaccine import from manufacturing facilities. However, there are limitations on the capacity of storage facilities at each level of the supply chain. The four warehouses located in London, Cardiff, Edinburgh, and Belfast have a maximum capacity of 150 m^3^, which is equivalent to 8.23 million doses. The twelve regional stores have maximum capacities of 90 m^3^, which is equivalent to 4.94 million doses. Lastly, the cluster of vaccination centres in Wales, Scotland, Northern Ireland, and the nine regions in England have storage capacities of 30 m^3^, equivalent to 1.65 million doses. Using this information, we estimated the installation and operating costs associated with each storage facility. For warehouses and regional stores, the installation costs are equivalent to $28,700 facility^−1^ week^−1^ and $17,200 facility^−1^ week^−1^ and the operating costs are $0.0098 dose^−1^ week^−1^ and $0.0129 dose^−1^ week^−1^, respectively. The facility installation cost takes into account the cost of building as well as the purchase cost of refrigerators and freezers. We assumed that cold chain equipment occupies about 70% of the total flow area of warehouses and regional stores. The remaining 30% is used for office equipment and space for installing ancillary devices. Operating costs comprise staff wages and the cost for the electricity required to power the cold chain equipment. The cost of quality control checks at warehouses is estimated using information provided by the National Institute for Biological Standard and Control (NIBSC) [67].

Vaccines are airlifted from plants to warehouses, while domestic distribution is carried out using either a refrigerated van or a refrigerated truck. The airfreight rate is about $3.26 kg^−1^ [68]. The maximum weight per trip is fixed at 21,000 kg [69], which is equivalent to 24.6 million doses per trip and $0.00278 per dose. Domestic transport by refrigerated van and refrigerated truck costs $0.10 km^−1^ and $0.26 km^−1^, respectively. These costs account for fuel, the annualised capital cost of vehicle, driver’s wages, and the average annual distance travelled by vehicle. Travel distances between supply chain entities were estimated using Google Maps, assuming straight lines between facilities, while travel times were calculated by dividing the travel distance by the average vehicle speed, as recommended by the Department of Transport UK [70].

Our case study investigates the supply and distribution of nucleic-acid-based SARS-CoV-2 vaccine candidate BNT162b2 developed and manufactured by Pfizer and BioNTech. Table S1 of Supplementary Materials summarises the characteristics of this vaccine.

## 3. Results and Discussions

### 3.1. Vaccine Demand Stratification

**Figure 4:**
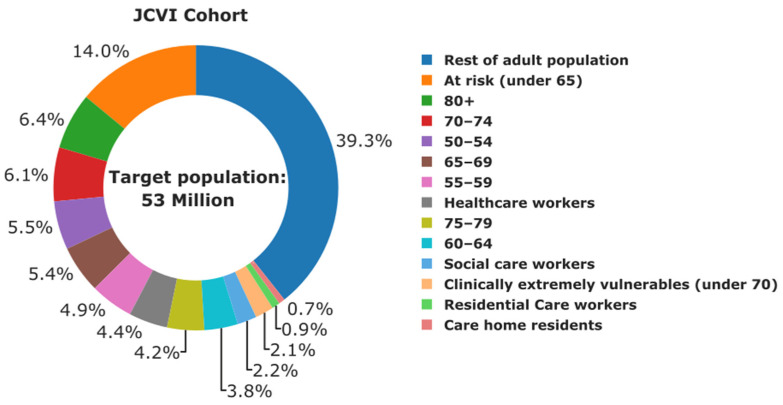
Target adult population in the UK and the distribution in accordance with cohorts recommended by the Joint Committee on Vaccination and Immunisation. Percentages are relative to the total target population.

Prior to conducting the COVID-19 vaccination campaign, the target population in a geographical region needs to be grouped and prioritised to increase the effectiveness of vaccination and to mitigate the impacts of infectious disease. According to the JCVI, the vaccination of the UK populace is being carried out in two phases. Phase 1 aims at direct prevention of mortality and focuses on vaccination of care home residents, residential care workers, those aged 80 plus, healthcare workers, social care workers, those aged 75–79, those aged 70–74, clinically extremely vulnerable individuals (under 70), those aged 65–69, at risk individuals (under 65), those aged 60–64, those aged 55–59, and those aged 50–54. Then, phase 2 aims to further reduce hospitalisation through vaccination of the rest of the adult population aged 18–49. Table S2 of Supplementary Materials shows the complete JCVI cohort classification into priority groups. This information together with the UK population data is used for inputs to the demand stratification model. Figure 4 shows the stratified UK population data according to the JCVI cohorts.

The target population corresponds to about 53 million individuals across the UK, out of which 39% are individuals aged 18–49. According to this baseline scenario, only healthy individuals aged below 18 are not considered. Apart from targeting the most vulnerable, vaccinating 80% of the UK population is needed to achieve herd immunity [71,72,73]. The results shown in Figure 4 indicate not only the target population but also provide a basis for estimating, in advance, the total doses required for the vaccination campaign and the number of doses allocated to each cohort. Knowing in advance the number of doses allocated to each cohort can allow government and policy-makers to set out an effective vaccination strategy that would focus on the most vulnerable individuals, especially when vaccines are under limited supply.

Figure 4 can be used together with Figure S1 of Supplementary Materials to understand regional vaccine demand within the UK based on cohorts recommended by JCVI. There is no significant variation in the distribution of care home residence and residential care workers across regions. A similar trend is observed in the distribution of clinically extremely vulnerable individuals under 70, social care workers, and individuals aged 75–79. By contrast, the distribution of the rest of adult population, individuals at risk under 65, individuals aged 80 and over, individuals aged 70–74, individuals aged 65–69, individuals aged 50–54, and individuals aged 55–59 varies visibly across regions.

Apart from visualising and estimating the demand of BNT162b2 SARS-CoV-2 vaccines in various regions within the UK, Figure S1 can be used to establish a supply plan for regions with the most vulnerable populations. Looking forward, the information in Figure S1 could also be used to estimate regional demand for booster vaccination campaigns targeted at vulnerable individuals.

### 3.2. Vaccine Administration

A vaccination campaign is always preceded by vaccine demand and workforce planning in order to meet the vaccination target. The vaccination timeframe required to vaccinate each cohort can be calculated by dividing the target population by the vaccination rate, where the latter takes into account staff working hours, time taken to vaccinate each patient, and daily vaccination target.

**Figure 5:**
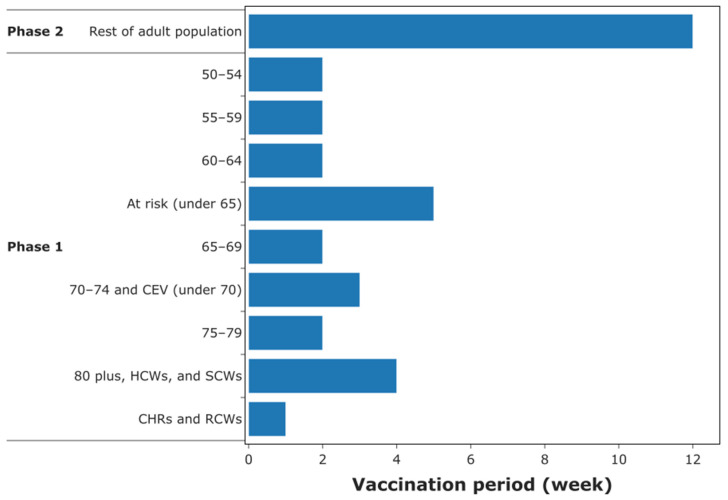
Timeframe required to vaccinate each JCVI cohort. CEV: clinically extremely vulnerable; HCWs: healthcare workers; SCWs: social care workers; CHRs: care home residences; RCWs: residential care workers.

Figure 5 shows the duration (in weeks) to vaccinate each cohort in the UK. Phase 1 of the vaccination exercise takes about 23 weeks, while phase 2 takes about 12 weeks. This indicates that the total duration of the vaccination exercise is about 38 weeks, considering that prime and booster doses are administered 3 weeks apart. Estimates of the duration required to vaccinate each JCVI cohort can be used to inform decision-making on when to start easing lockdown restrictions as vaccination progresses, especially when the high-risk groups have been vaccinated.

A successful vaccination campaign requires a sufficient number of healthcare workers. The number of healthcare workers required for the vaccination exercise across the regions is considered. The overall staff requirement numbers across the UK range between 23,896 and 97,466. London requires the highest number of healthcare workers (range: 2068–15,986), whilst Northern Ireland has the lowest staff requirements (range: 800–3627). Staff requirements in other regions are as follows: North East (range: 965–4076), North West (range: 2498–10,759), Yorkshire and the Humber (range: 1900–8144), East Midlands (range: 1690–7214), West Midlands (range: 2077–8862), East of England (range: 2382–9883), South East (range: 3488–14,497), South West (range: 2377–9567), Wales (range: 1118–5084), and Scotland (range: 2083–11,222).

Ahead of a COVID-19 vaccination campaign, the estimated number of healthcare workers can be used by government and policy-makers to assess workforce requirements. For example, by comparing the staff requirement estimates against the existing workforce across regions in the UK, it is possible to identify regions with shortfalls and take measures by recruiting additional staff or re-directing staff from regions with surplus workforce. In addition, knowing the workforce requirements in advance can ensure efficient financial planning, thereby keeping staff wages within an overall budget. Recall that the results presented in Figure 5 assumes that each staff member works a six-hour shift and spends at most ten minutes to vaccinate each patient (including screening, obtaining consent, etc.), meaning results will vary for different work patterns.

To meet the COVID-19 vaccination target of 500,000 patients per day across the UK, it is necessary to vaccinate between 860,250 and 3,508,792 individuals on a weekly basis. The largest weekly vaccination target is observed in London (range: 74,450–575,482), while the lowest is observed in Northern Ireland (range: 28,800–130,583). Weekly vaccination targets in the other regions are as follows: North east (range: 34,753–146,729), North west (range: 89,914–387,321), Yorkshire and the Humber (range: 68,390–293,166), East Midlands (range: 60,827–259,706), West Midlands (range: 74,769–319,050), East of England (range: 85,753–355,800), South East (range: 125,574–521,877), South West (range: 85,571–344,416), Wales (range: 40,250–183,017), and Scotland (range: 75,000–404,000). Naturally, regions with large numbers of individuals to be vaccinated require large numbers of HCWs, and vice versa. The weekly vaccinations give an indication about the capacity of vaccine administration points that would be needed in Wales, Scotland, Northern Ireland, and the nine regions in England.

A vaccination campaign may not proceed without sufficient supply of vaccines from manufacturing plants. Using information on weekly vaccination targets and vaccine doses needed to fully immunise each patient (in this case 2 doses administered 3 weeks apart), the vaccine demand profile during a COVID-19 vaccination campaign in the UK can be estimated.

**Figure 6:**
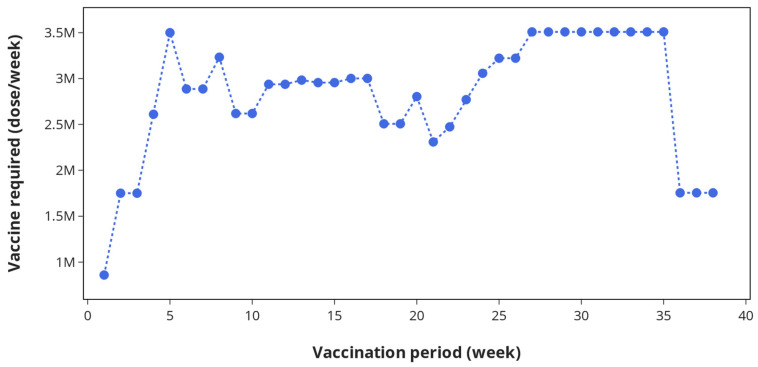
Vaccine demand profile showing the number of doses required each week to satisfy demand at vaccination centres in the UK.

Figure 6 shows the number of doses that should be made available per week in the UK to meet the timeframe set out in Figure 5. The demand for the BNT162b2 SARS-CoV-2 vaccine varies on a weekly basis throughout the vaccination period and ranges between 0.86 and 3.51 million doses per week. This demand profile can serve as an important tool not only for guiding government and policy-makers on securing contracts for vaccine supply from manufacturers or contract manufacturing organisations, but also for developing supply plans during the vaccination period to meet the target of vaccinating up to 500,000 patients per day. Over-supply could lead to vaccine wastage, which can have a significant cost implication, while low supply can lead to shortfalls, which can lead to a surge in infection. The vaccine demand profile can also be used in designing a new vaccine supply chain or re-designing an existing supply chain to meet the COVID-19 vaccination target across the UK.

### 3.3. Vaccine Supply Chain Optimisation

Using the demand profile shown in Figure 6, information presented in Section 3.3, and the supply chain model in Section 3.2, an optimisation study is carried out for the supply chain in Figure 3 to meet COVID-19 vaccination target of up to 500,000 patients per day across the UK. To investigate the impacts of vaccine shortage on logistics and vaccine administration, a bi-objective optimisation model [74] is set up considering the logistics cost and backlog as objectives. The accumulation of backlog as a result of vaccine shortage can impact the logistics cost and logistics cost per fully immunised patient (FIP), as well as vaccine availability in England, Scotland, Wales, and Northern Ireland. 

**Figure 7:**
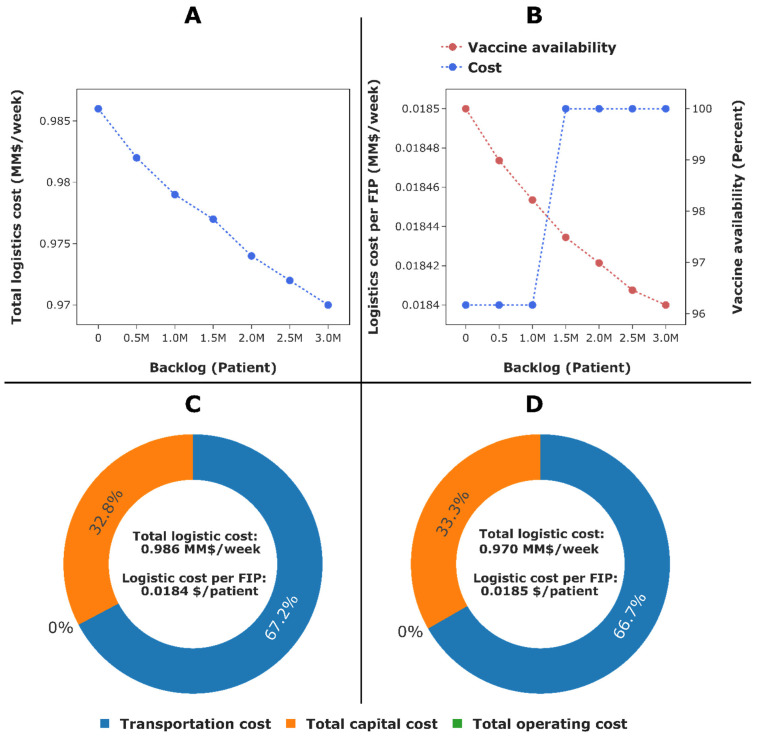
Outputs from vaccine supply chain optimisation. (**A**) Pareto frontier for the bi-objective optimisation model, considering the total logistics cost and backlog as objectives. The upper extreme point indicates the supply chain design without vaccine shortage (Scenario 1), while the lower extreme point indicates the design with lowest total logistics cost (Scenario 2). (**B**) Impact of backlog on logistics cost per fully immunised patient (blue dotted curve) and vaccine availability (the red dotted curve). (**C**,**D**) Total logistics cost required to deliver vaccines from manufacturing plant in Puur, Belgium, to administration points in all regions across the UK, vaccine procurement excluded, in scenarios 1 (**C**) and 2 (**D**). FIP: fully immunised patient.

The supply chain optimisation results are illustrated on Figure 7 and further discussed below. The flow of BNT162b2 SARS-CoV-2 vaccines from the factory in Puur, Belgium, to warehouses in London, Cardiff, Edinburgh, and Belfast is shown in Figure S2 of Supplementary Materials; the quality control capacity required at warehouses across the UK in Figure S3; the weekly flow of vaccine shippers from warehouses to vaccination centres and back to warehouses in Figure S4; and the total trips covered by transportation modes at various levels of the supply chain in Figure S5.

The Pareto frontier shown in Figure 7A comprises non-dominated points in the bi-objective optimisation model. For these points, it is not possible to make improvement to the total logistics cost without worsening backlog, and vice versa. Each point on the Pareto frontier represents an alternative vaccine supply chain design and its associated operating strategy. Clearly, an increase in backlog is accompanied by a reduction in the total logistics cost, and vice versa. A zero backlog means that all target individuals in England, Scotland, Wales, and Northern Ireland have received their prime and booster jabs, leading to 100% vaccine availability (upper extreme point of the vaccine availability curve in Figure 7B). The impact of backlog on vaccine availability is quite pronounced. However, accumulation of backlog during the COVID-19 vaccination campaign is not predicted to significantly reduce the logistics cost or logistics cost per FIP.

Figure 7C,D present the total logistics costs for the delivery of the BNT162b2 SARS-CoV-2 vaccine from the factory in Puur, Belgium, to administration points in England, Scotland, Wales, and Northern Ireland. No operating cost is observed in scenarios 1 or 2 because vaccines are stored at clinics and vaccination centres only, with no inventories at warehouses or regional vaccine stores. As expected, the total annualised logistics cost obtained in scenario 2, $0.970 million week^−1^, is lower than that of scenario 1, $0.986 million week^−1^. However, this does not translate into a significant difference in logistics cost per FIP for scenarios 1 ($0.0184 patient^−1^) and 2 ($0.0185 patient^−1^). Recall that these estimates are calculated considering JCVI cohorts only and may vary if new cohorts for individuals aged 17 years and under are added. It is also worth reiterating that the total annualised logistics cost excludes the cost of vaccine shippers, cost of quality control checks at warehouses, and cost of vaccine procurement.

Besides the total logistics cost and logistics cost per FIP, additional key performance measures reported in Table 1 include the costs of shippers, dry ice, vaccine procurement, quality control, and vaccinator wages. The costs of vaccinating individuals differ in scenarios 1 and 2, since the total numbers of individuals to be vaccinated are not the same as a result of backlog. Similarly, the cost of vaccine procurement in scenario 1 is 2% ($40 million) greater than in scenario 2. The extra cost observed in scenario 1 is due to the supply of sufficient doses of BNT162b2 vaccines to vaccination centres across the UK (see Section 3.4). The increase in the vaccine procurement cost is also reflected in the cost of dry ice, since additional dry ice would be required to keep the vaccine at −80 ℃ during transportation. The extra dry ice needed is approximately 11 ton when compared to scenario 2. The cost of purchasing thermal shippers in scenario 2 is 5% ($0.330 million) lower than in scenario 1. This results from the fact that the numbers of shippers required are 1160 and 1110 in scenarios 1 and 2, respectively.

From a logistics point of view, handling a small number of thermal shippers can lead to more efficient management, as fewer shippers would be recycled during the vaccination campaign. Furthermore, less workforce would be required to manage a small number of shippers, leading to savings in staff wages. On the contrary, handling a large number of thermal shippers can increase the transportation cost (and consequently total logistics cost) as a result of the large payload that needs to be transported from warehouses to administration points. The increase in total logistics cost can be exacerbated by capital investment in cold chain equipment required to store vaccines and shippers at warehouses, regional stores, and administration points. Regardless of the scenario, the cost of vaccine procurement dominates the overall cost, followed by wages paid to vaccinate all target individuals in England, Scotland, Wales, and Northern Ireland. Therefore, to achieve additional cost savings, the key targets for improvement are the vaccine procurement cost and vaccinator wages. The cost of administering vaccines to individuals is fixed at $17.10 (£12.80) per patient, plus $13.36 (£10) when vaccines are administered in care homes. Hence, one option to minimise the total vaccinator wages is that government needs to either re-negotiate the unit price for administering vaccines or consider other alternatives, for example by training and using the military or paramedics for the vaccination campaign.

The selling price of the BNT162b2 SARS-CoV-2 vaccine differs considerably across the world, ranging between $7 and $20 per dose [32], with the UK government paying $18.66 per dose. Considering the global selling price of BNT162b2 SARS-CoV-2 vaccines, the cost of procuring 107 million doses (UK demand) can vary significantly—between MM$ 750.4 and $2.14 billion, which corresponds to a 62.5% decrease and 7.2% increase when compared to the current price the UK government is paying to procure the BNT162b2 SARS-CoV-2 vaccines. As well as re-negotiating the unit price per dose with vaccine manufacturers, another cost saving option is to use cheaper vaccines, such as the AZD1222 SARS-CoV-2 vaccine sold for between $3 and $5 per dose [32]. Even though the cost of vaccine procurement is high, it is of course a cheaper option compared to the economic, social, and health impacts of coronavirus. Over one year, the lockdowns imposed by the UK government to reduce the transmission of coronavirus have cost the UK economy in excess of $334 (£251) billion [75]. This cost is greater than the combined cost of vaccine procurement, vaccinator wages, vaccine thermal shippers, dry ice, quality control checks, and logistics.

### 3.4. COVID-19 Vaccines Administered at Vaccination Centres

**Figure 8:**
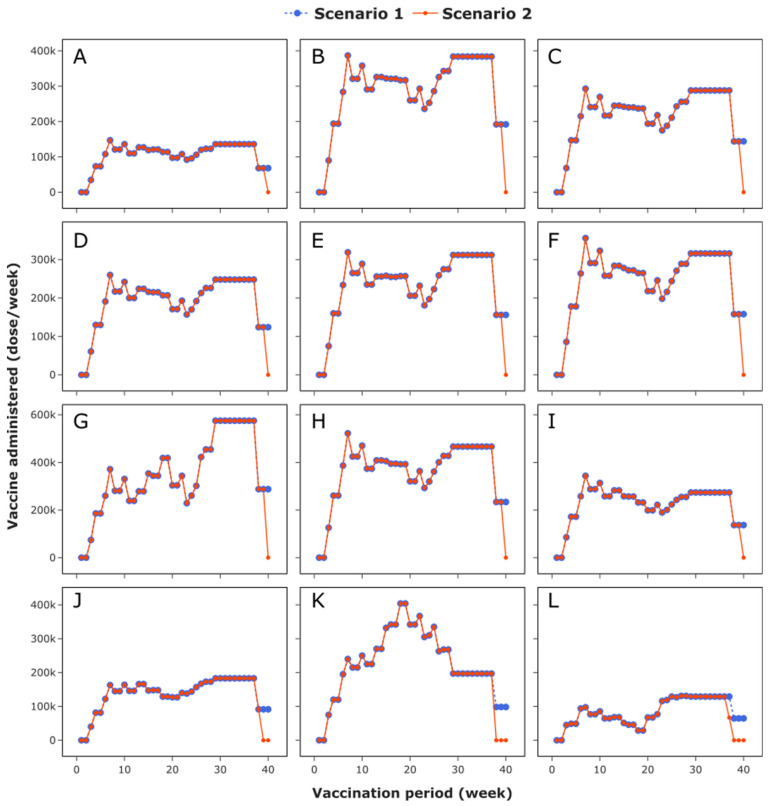
Vaccine administration at various locations across the UK: (**A**) North East; (**B**) North West; (**C**) Yorkshire and the Humber; (**D**) East Midlands; (**E**) West Midlands; (**F**) East of England; (**G**) London; (**H**) South East; (**I**) South West; (**J**) Wales; (**K**) Scotland; (**L**) Northern Ireland. The blue dotted curve denotes scenario 1, whilst the red solid curve denotes scenario 2.

In this case study, the daily vaccination rate across the UK can rise up to 500,000 patients per day. Figure 8 shows the weekly vaccines administered to all JCVI cohorts in Scotland, Wales, Northern Ireland, and the nine regions in England. Note the absence of vaccination in week 1 and week 2, as the initial batches of BNT162b2 SARS-CoV-2 vaccine undergo quality control checks during this period.

Vaccination starts in week 3 with the care home residents, followed by residential care workers, individuals aged 80 +, healthcare workers, social care workers, individuals aged 75–79, individuals aged 70–74, clinically extremely vulnerable individuals (under 70), individuals aged 65–69, at risk individuals (under 65), individuals aged 60–64, individuals aged 55–59, individuals aged 50–54, and the rest of the adult population. The variation in the vaccination profile across regions reflects the heterogeneity in age distribution in the UK. The increase in the administration rate observed in week 6 is due to individuals coming to vaccination centres for their second or booster jab. Therefore, vaccination centres should have enough capacity to accommodate the highest weekly vaccination target. In both scenarios 1 and 2, the required capacity rates for vaccination centres in the North East, North West, Yorkshire and the Humber, East Midlands, West Midlands, East of England, London, South East, South West, Wales, Scotland, and Northern Ireland should be at least 0.147, 0.387, 0.293, 0.259, 0.319, 0.356, 0.575, 0.522, 0.344, 0.183, 0.404, and 0.131 million doses per week, respectively.

For all vaccination centres in scenario 1, logistics optimisation ensures that enough vaccines are available to vaccinate the entire target population, leading to 100% vaccine availability. However, in scenario 2, a shortage in vaccine supply leads to a backlog in vaccine administration points, resulting in a drop in vaccine availability to 97.9% (see Figure 7B). The drop in vaccine availability is reflected in vaccination centres across the UK, as shown in Figure 8. The largest shortage is observed in vaccination centres located in Northern Ireland and Scotland, where stockouts of four weeks and three weeks are observed, whereas in Wales, the North East, and Yorkshire and the Humber, the stockout duration drops to two weeks. The remaining regions observe stockout periods of one week only. The variation in stockout duration across the UK is caused by the differences in distance between plants, warehouses, regional stores, and administration points. Since the warehouse located in London is much closer to the plant in Puur, Belgium, the logistics optimisation first satisfies the weekly demand in this location, followed by warehouses in Wales, Scotland, and Northern Ireland. Similarly, within England, the logistics optimisation first satisfies the weekly demand for locations closer to the warehouse in London (see Figure 8). This results from the fact that the transportation cost dominants the logistics cost (see Figure 7C,D); therefore, to minimise the logistics cost, the number of trips to the farthest locations within the vaccine distribution network needs to be minimised.

The stockouts observed in vaccination centres across the UK imply that no vaccination is conducted during these periods. In Figure 8, the stockout mostly affects individuals aged 18–49 arriving at vaccination centres to receive their second or booster jabs. Even though this cohort is less vulnerable to COVID-19 and the first or prime dose provides a high protection against the novel coronavirus in addition to a reduction in transmission, it is important to ensure that anyone willing to take the vaccine is fully immunised. This practice can increase trust and confidence in the immunisation programme, leading to a high acceptability rate and a reduction in vaccine hesitancy. Figure 8 demonstrates the capabilities of the proposed vaccine distribution and administration decision support tool, not only in monitoring the progress of a vaccination, but also in identifying regions, locations, and cohorts that are likely to be affected should there be a shortage in vaccine supply during the COVID-19 vaccination campaign.

## 4. Conclusions

We have developed a new optimisation-based vaccine supply chain model and embedded this model within a systematic framework that supports the planning and delivery of a vaccination campaign against infectious diseases. The framework consists of three main steps. Step 1 (demand stratification) uses information on the geographical population and cohort size to generate the stratified vaccine demand. Step 2 (vaccine administration) utilises data on staff working hours and vaccination targets together with the stratified demand from step 1 to estimate the vaccination timeframe, workforce needed, and individuals that must be vaccinated to meet the vaccination target. Finally, step 3 (vaccine supply and delivery) utilises outputs from steps 1 and 2 to predict the logistics cost and plan the distribution and administration of vaccines to targeted individuals. Optimisation of COVID-19 vaccine supply chain indicates that minimisation of the total logistics cost can lead to a cost-optimal vaccine supply chain that ensures a high vaccine availability at administration points. Regardless of whether a backlog exists or not, the transportation cost dominates the total annualised logistics cost, which can be attributed to the short shelf life of the BNT162b2 SARS-CoV-2 vaccine. Furthermore, the estimated total cost of supply chain components (thermal shippers, dry ice, staff wages, vaccine procurement, and quality control checks) indicates that the cost of vaccine procurement dominates, followed by vaccinator wages. Analysis of the impacts of backlog on other key performance indicators by applying bi-objective optimisation indicates that an increase in backlog due to shortage in vaccine supply or failure to meet vaccination targets does not have a significant impact on the logistics cost and logistics cost per fully immunised patient, but can lead to a reduction in vaccine availability (from 100 to 96 percent) in England, Scotland, Wales, and Northern Ireland. This reduction in vaccine availability affects vaccination coverage, thereby increasing local transmission of COVID-19, which can lead to a surge in the number of infections, increased hospitalisation, and increased death rates across the UK.

## Figures and Tables

**Table 1:** Estimated cost of vaccine supply chain components such as vaccine thermal shippers, dry ice, vaccinator wages, vaccine procurement, and quality control checks.

Item.	Scenario 1	Scenario 2	Units
Cost of vaccine shipper	7.72	7.39	million $
Cost of dry ice	20.30	19.90	million $
Cost of vaccinating individuals	1.88	1.85	billion $
Cost of vaccinating individuals at care home	24.10	24.10	million $
Cost of vaccine procured	2.00	1.96	billion $
Cost of quality control checks	59.90	58.70	million $

## Supplementary Materials

The following are available online at https://www.mdpi.com/article/10.3390/vaccines9121460/s1, Supplementary 1: Supplementary result; Supplementary 2: Information related to vaccine characteristics and vaccine administration; Supplementary 3: Details of supply chain modelling; Supplementary 4: Modelling vaccine administration and demand stratification.

## References

[1] WHO Timeline—COVID-19. https://www.who.int/news/item/27-04-2020-who-timeline—covid-19.

[2] Asselah T., Durantel D., Pasmant E., Lau G., Schinazi R.F. (2021). COVID-19: Discovery, Diagnostics and Drug Development. J. Hepatol..

[3] Cucinotta D., Vanelli M. (2020). WHO Declares COVID-19 a Pandemic. Acta Biomed..

[4] Liu Y.C., Kuo R.L., Shih S.R. (2020). COVID-19: The First Documented Coronavirus Pandemic in History. Biomed. J..

[5] Cepi Advances 8 COVID-19 Vaccine Programmes. https://www.glopid-r.org/newsletter-13th-edition/cepi-advances-8-covid-19-vaccine-programmes.

[6] Why We’re Giving $250 Million More to Fight COVID-19. https://www.gatesfoundation.org/ideas/articles/coronavirus-funding-additional-250-million-suzman.

[7] Global Partnership to Make Available 120M Affordable, Quality COVID-19 Rapid Tests for Low- and Middle-Income Countries. https://www.clintonhealthaccess.org/global-partnership-to-make-available-120m-affordable-quality-covid-19-rapid-tests-for-low-and-middle-income-countries.

[8] Funding COVID-19 Vaccines: A Timeline. https://www.devex.com/news/funding-covid-19-vaccines-a-timeline-97950.

[9] Voysey M., Clemens S.A.C., Madhi S.A., Weckx L.Y., Folegatti P.M., Aley P.K., Angus B., Baillie V.L., Barnabas S.L., Bhorat Q.E. (2021). Safety and Efficacy of the ChAdOx1 NCoV-19 Vaccine (AZD1222) against SARS-CoV-2: An Interim Analysis of Four Randomised Controlled Trials in Brazil, South Africa, and the UK. Lancet.

[10] Hung I.F.N., Poland G.A. (2021). Single-Dose Oxford—AstraZeneca COVID-19 Vaccine Followed by a 12-Week Booster. Lancet.

[11] Sadoff J., Le Gars M., Shukarev G., Heerwegh D., Truyers C., de Groot A.M., Stoop J., Tete S., Van Damme W., Leroux-Roels I. (2021). Interim Results of a Phase 1–2a Trial of Ad26.COV2.S Covid-19 Vaccine. N. Engl. J. Med..

[12] Tanne J.H. (2021). Covid-19: US Authorises Johnson and Johnson Vaccine Again, Ending Pause in Rollout. BMJ.

[13] Livingston E.H., Malani P.N., Creech C.B. (2021). The Johnson & Johnson Vaccine for COVID-19. JAMA J. Am. Med. Assoc..

[14] Baraniuk C. (2021). Covid-19: What Do We Know about Sputnik v and Other Russian Vaccines?. BMJ.

[15] Logunov D.Y., Dolzhikova I.V., Zubkova O.V., Tukhvatullin A.I., Shcheblyakov D.V., Dzharullaeva A.S., Grousova D.M., Erokhova A.S., Kovyrshina A.V., Botikov A.G. (2020). Safety and Immunogenicity of an RAd26 and RAd5 Vector-Based Heterologous Prime-Boost COVID-19 Vaccine in Two Formulations: Two Open, Non-Randomised Phase 1/2 Studies from Russia. Lancet.

[16] Logunov D.Y., Dolzhikova I.V., Shcheblyakov D.V., Tukhvatulin A.I., Zubkova O.V., Dzharullaeva A.S., Kovyrshina A.V., Lubenets N.L., Grousova D.M., Erokhova A.S. (2021). Safety and Efficacy of an RAd26 and RAd5 Vector-Based Heterologous Prime-Boost COVID-19 Vaccine: An Interim Analysis of a Randomised Controlled Phase 3 Trial in Russia. Lancet.

[17] Walsh E.E., Frenck R.W., Falsey A.R., Kitchin N., Absalon J., Gurtman A., Lockhart S., Neuzil K., Mulligan M.J., Bailey R. (2020). Safety and Immunogenicity of Two RNA-Based Covid-19 Vaccine Candidates. N. Engl. J. Med..

[18] Polack F.P., Thomas S.J., Kitchin N., Absalon J., Gurtman A., Lockhart S., Perez J.L., Pérez Marc G., Moreira E.D., Zerbini C. (2020). Safety and Efficacy of the BNT162b2 MRNA Covid-19 Vaccine. N. Engl. J. Med..

[19] Lamb Y.N. (2021). BNT162b2 MRNA COVID-19 Vaccine: First Approval. Drugs.

[20] Anderson E.J., Rouphael N.G., Widge A.T., Jackson L.A., Roberts P.C., Makhene M., Chappell J.D., Denison M.R., Stevens L.J., Pruijssers A.J. (2020). Safety and Immunogenicity of SARS-CoV-2 MRNA-1273 Vaccine in Older Adults. N. Engl. J. Med..

[21] Baden L.R., El Sahly H.M., Essink B., Kotloff K., Frey S., Novak R., Diemert D., Spector S.A., Rouphael N., Creech C.B. (2021). Efficacy and Safety of the MRNA-1273 SARS-CoV-2 Vaccine. N. Engl. J. Med..

[22] Kim J.H., Marks F., Clemens J.D. (2021). Looking beyond COVID-19 Vaccine Phase 3 Trials. Nat. Med..

[23] Rauch S., Roth N., Schwendt K., Fotin-Mleczek M., Mueller S.O., Petsch B. (2021). MRNA-Based SARS-CoV-2 Vaccine Candidate CVnCoV Induces High Levels of Virus-Neutralising Antibodies and Mediates Protection in Rodents. NPJ Vaccines.

[24] The Sinopharm COVID-19 Vaccine: What You Need to Know. https://www.who.int/news-room/feature-stories/detail/the-sinopharm-covid-19-vaccine-what-you-need-to-know.

[25] Shinde V., Bhikha S., Hoosain Z., Archary M., Bhorat Q., Fairlie L., Lalloo U., Masilela M.S.L., Moodley D., Hanley S. (2021). Efficacy of NVX-CoV2373 Covid-19 Vaccine against the B.1.351 Variant. N. Engl. J. Med..

[26] Mahase E. (2021). Covid-19: Novavax Vaccine Efficacy Is 86% against UK Variant and 60% against South African Variant. BMJ.

[27] West S., Kis Z., Kontoravdi C., Papathanasiou M., Shah N., Chachuat B. Is the World Ready to Produce a Billion Doses of a COVID-19 Vaccine?. https://www.imperial.ac.uk/news/197321/is-world-ready-produce-billion-doses.

[28] Q&A: Cold Chains, COVID-19 Vaccines and Reaching Low-Income Countries. https://www.imperial.ac.uk/news/209993/qa-cold-chains-covid-19-vaccines-reaching.

[29] Acharya K.P., Ghimire T.R., Subramanya S.H. (2021). Access to and Equitable Distribution of COVID-19 Vaccine in Low-Income Countries. NPJ Vaccines.

[30] Hall S., Kaplow L., Sun Y.S., Holt T.Z. ‘None Are Safe until All Are Safe’: COVID-19 Vaccine Rollout in Low- and Middle-Income Countries. https://www.mckinsey.com/industries/healthcare-systems-and-services/our-insights/none-are-safe-until-all-are-safe-covid-19-vaccine-rollout-in-low-and-middle-income-countries.

[31] Choi E.M. (2021). COVID-19 Vaccines for Low- and Middle-Income Countries. Trans. R. Soc. Trop. Med. Hyg..

[32] Wouters O.J., Shadlen K.C., Salcher-Konrad M., Pollard A.J., Larson H.J., Teerawattananon Y., Jit M. (2021). Challenges in Ensuring Global Access to COVID-19 Vaccines: Production, Affordability, Allocation, and Deployment. Lancet.

[33] WHO (2020). Framework for Decision-Making: Implementation of Mass Vaccination Campaigns in the Context of COVID-19.

[34] Becker A.D., Grantz K.H., Hegde S.T., Bérubé S., Cummings D.A.T., Wesolowski A. (2021). Development and Dissemination of Infectious Disease Dynamic Transmission Models during the COVID-19 Pandemic: What Can We Learn from Other Pathogens and How Can We Move Forward?. Lancet Digit. Health.

[35] Alvarez M.M., González-González E., Trujillo-de Santiago G. (2021). Modeling COVID-19 Epidemics in an Excel Spreadsheet to Enable First-Hand Accurate Predictions of the Pandemic Evolution in Urban Areas. Sci. Rep..

[36] Bertozzi A.L., Franco E., Mohler G., Short M.B., Sledge D. (2020). The Challenges of Modeling and Forecasting the Spread of COVID-19. Proc. Natl. Acad. Sci. USA.

[37] James L.P., Salomon J.A., Buckee C.O., Menzies N.A. (2021). The Use and Misuse of Mathematical Modeling for Infectious Disease Policymaking: Lessons for the COVID-19 Pandemic. Med. Decis. Mak..

[38] Carcione J.M., Santos J.E., Bagaini C., Ba J. (2020). A Simulation of a COVID-19 Epidemic Based on a Deterministic SEIR Model. Front. Public Health.

[39] Thompson R.N. (2020). Epidemiological Models Are Important Tools for Guiding COVID-19 Interventions. BMC Med..

[40] Kis Z., Kontoravdi C., Shattock R., Shah N. (2021). Resources, Production Scales and Time Required for Producing RNA Vaccines for the Global Pandemic Demand. Vaccines.

[41] Van de Berg D., Kis Z., Behmer C.F., Samnuan K., Blakney A.K., Kontoravdi C., Shattock R., Shah N. (2021). Quality by Design Modelling to Support Rapid RNA Vaccine Production against Emerging Infectious Diseases. NPJ Vaccines.

[42] Kis Z., Kontoravdi C., Dey A.K., Shattock R., Shah N. (2020). Rapid Development and Deployment of High-Volume Vaccines for Pandemic Response. J. Adv. Manuf. Process..

[43] Reader D., Li J., McDonnel A., Yadav P. Modelling the Manufacturing Process for COVID-19 Vaccines: Our Approach. https://www.cgdev.org/blog/modelling-manufacturing-process-covid-19-vaccines-our-approach.

[44] Modelling of Manufacturing COVID-19 Vaccines. https://www.brydenwood.co.uk/projects/modelling-of-manufacturing-covid19-vaccines/s101877.

[45] Kis Z. Enhancing Vaccine Platforms: Computational Models Accelerate Development, Manufacturing, and Distribution. https://bioprocessintl.com/manufacturing/vaccines/enhancing-vaccine-platforms-a-computational-modeling-framework-accelerates-development-manufacturing-and-distribution.

[46] Guignard A., Praet N., Jusot V., Bakker M., Baril L. (2019). Introducing New Vaccines in Low- and Middle-Income Countries: Challenges and Approaches. Expert Rev. Vaccines.

[47] Lee B.Y., Assi T.M., Rajgopal J., Norman B.A., Chen S.I., Brown S.T., Slayton R.B., Kone S., Kenea H., Welling J.S. (2012). Impact of Introducing the Pneumococcal and Rotavirus Vaccines into the Routine Immunization Program in Niger. Am. J. Public Health.

[48] Haidari L.A., Wahl B., Brown S.T., Privor-Dumm L., Wallman-Stokes C., Gorham K., Connor D.L., Wateska A.R., Schreiber B., Dicko H. (2015). One Size Does Not Fit All: The Impact of Primary Vaccine Container Size on Vaccine Distribution and Delivery. Vaccine.

[49] Brown S.T., Schreiber B., Cakouros B.E., Wateska A.R., Dicko H.M., Connor D.L., Jaillard P., Mvundura M., Norman B.A., Levin C. (2014). The Benefits of Redesigning Benin’s Vaccine Supply Chain. Vaccine.

[50] Lee B.Y., Schreiber B., Wateska A.R., Connor D.L., Dicko H.M., Jaillard P., Mvundura M., Levin C., Avella M., Haidari L.A. (2015). The Benin Experience: How Computational Modeling Can Assist Major Vaccine Policy Changes in Low- and Middle-Income Countries. Vaccine.

[51] Brown S.T., Lee B.Y. (2014). Unless Changes Are Made in Benin, Multiple Storage and Transport Bottlenecks May Prevent Vaccines from Reaching the Population. Vaccine.

[52] De Carvalho M.I., Ribeiro D., Barbosa-Povoa A.P., Barbosa-Povoa A., Jenzer H., de Miranda J. (2019). Design and Planning of Sustainable Vaccine Supply Chain. Pharmaceutical Supply Chains—Medicines Shortages.

[53] Sadjadi S.J., Ziaei Z., Pishvaee M.S. (2019). The Design of the Vaccine Supply Network under Uncertain Condition: A Robust Mathematical Programming Approach. J. Model. Manag..

[54] Chen S.I., Norman B.A., Rajgopal J., Assi T.M., Lee B.Y., Brown S.T. (2014). A Planning Model for the WHO-EPI Vaccine Distribution Network in Developing Countries. IIE Trans..

[55] Kis Z., Papathanasiou M., Calvo-Serrano R., Kontoravdi C., Shah N. (2019). A Model-Based Quantification of the Impact of New Manufacturing Technologies on Developing Country Vaccine Supply Chain Performance: A Kenyan Case Study. J. Adv. Manuf. Process..

[56] Georgiadis G.P., Georgiadis M.C. (2021). Optimal Planning of the COVID-19 Vaccine Supply Chain. Vaccine.

[57] Office for National Statistics. https://www.ons.gov.uk.

[58] (2020). Joint Committee on Vaccination and Immunisation: Advice on Priority Groups for COVID-19 Vaccination. https://www.gov.uk/government/publications/priority-groups-for-coronavirus-covid-19-vaccination-advice-from-the-jcvi-30-december-2020/joint-committee-on-vaccination-and-immunisation-advice-on-priority-groups-for-covid-19-vaccination-30-december-2020.

[59] Crocker-Buque T., Mohan K., Ramsay M., Edelstein M., Mounier-Jack S. (2019). What Is the Cost of Delivering Routine Vaccinations at GP Practices in England? A Comparative Time-Driven Activity-Based Costing Analysis. Hum. Vaccines Immunother..

[60] Crocker-Buque T., Mounier-Jack S. (2018). Vaccination in England: A Review of Why Business as Usual Is Not Enough to Maintain Coverage. BMC Public Health.

[61] Crocker-Buque T., Edelstein M., Mounier-Jack S. (2018). A Process Evaluation of How the Routine Vaccination Programme Is Implemented at GP Practices in England. Implement. Sci..

[62] UK Hits Nearly 500,000 Covid Vaccinations a Day as Three-Quarters of Over-80s Now Jabbed. https://www.thesun.co.uk/news/uknews/13836401/uk-hits-nearly-500000-covid-vaccinations-a-day.

[63] Covid Vaccine: How Many People in the UK Have Been Vaccinated So Far?. https://www.bbc.co.uk/news/health-55274833.

[64] Coronavirus (COVID-19) in the UK. https://coronavirus.data.gov.uk/details/vaccinations.

[65] COVID-19 Vaccination Programme. https://www.bma.org.uk/advice-and-support/covid-19/vaccines/covid-19-vaccination-programme.

[66] Vaccination Sites. https://www.england.nhs.uk/coronavirus/publication/vaccination-sites.

[67] NIBSC Statement on Batch Testing for the COVID-19 Vaccine AstraZeneca. https://www.nibsc.org/about_us/latest_news/batch_testing.aspx.

[68] Air Freight & Air Cargo Shipping: Air Freight Charges, Rates, Costs & Quotes. https://www.freightos.com/freight-resources/air-freight-rates-cost-prices.

[69] World Class Shipping-New York. http://www.worldclassshipping.com/aircraft.html.

[70] Department for Transport. https://assets.publishing.service.gov.uk/government/uploads/system/uploads/attachment_data/file/782192/background-quality-report.pdf.

[71] COVID-19: UK Set to Reach Herd Immunity “Milestone” Within Days, Say Scientists. https://news.sky.com/story/covid-19-uk-set-to-reach-herd-immunity-milestone-within-days-say-scientists-12269405.

[72] How Many People Have COVID-19 Antibodies in the UK?. https://www.statista.com/chart/23961/uk-share-with-covid-antibodies.

[73] Herd Immunity: Can the UK Get There?. https://theconversation.com/herd-immunity-can-the-uk-get-there-160026.

[74] Emmerich M.T.M., Deutz A.H. (2018). A Tutorial on Multiobjective Optimization: Fundamentals and Evolutionary Methods. Nat. Comput..

[75] A Year of Covid Lockdowns Has Cost the UK Economy £251bn, Study Says. https://www.theguardian.com/business/2021/mar/22/a-year-of-covid-lockdowns-has-cost-the-uk-economy-251bn-study-says.

