# Supplementary material for: Model-Based Planning and Delivery of Mass Vaccination Campaigns against Infectious Disease: Application to the COVID-19 Pandemic in the UK
Source: Vaccines (Basel). 2021 Dec 10;9(12):1460. doi: 10.3390/vaccines9121460 (PMC8706890; doi:10.3390/vaccines9121460)
Supplement: Supplementary file 1 [file vaccines-09-01460-s001.zip › vaccines-1472780-supplementary.pdf]

Supplementary material

Supplementary 1: Supplementary results

S1.1 Vaccine demand stratification

The outcome from the demand stratification studies is shown in Figure S1, where each panel represents the distribution of JCVI cohorts across the UK.

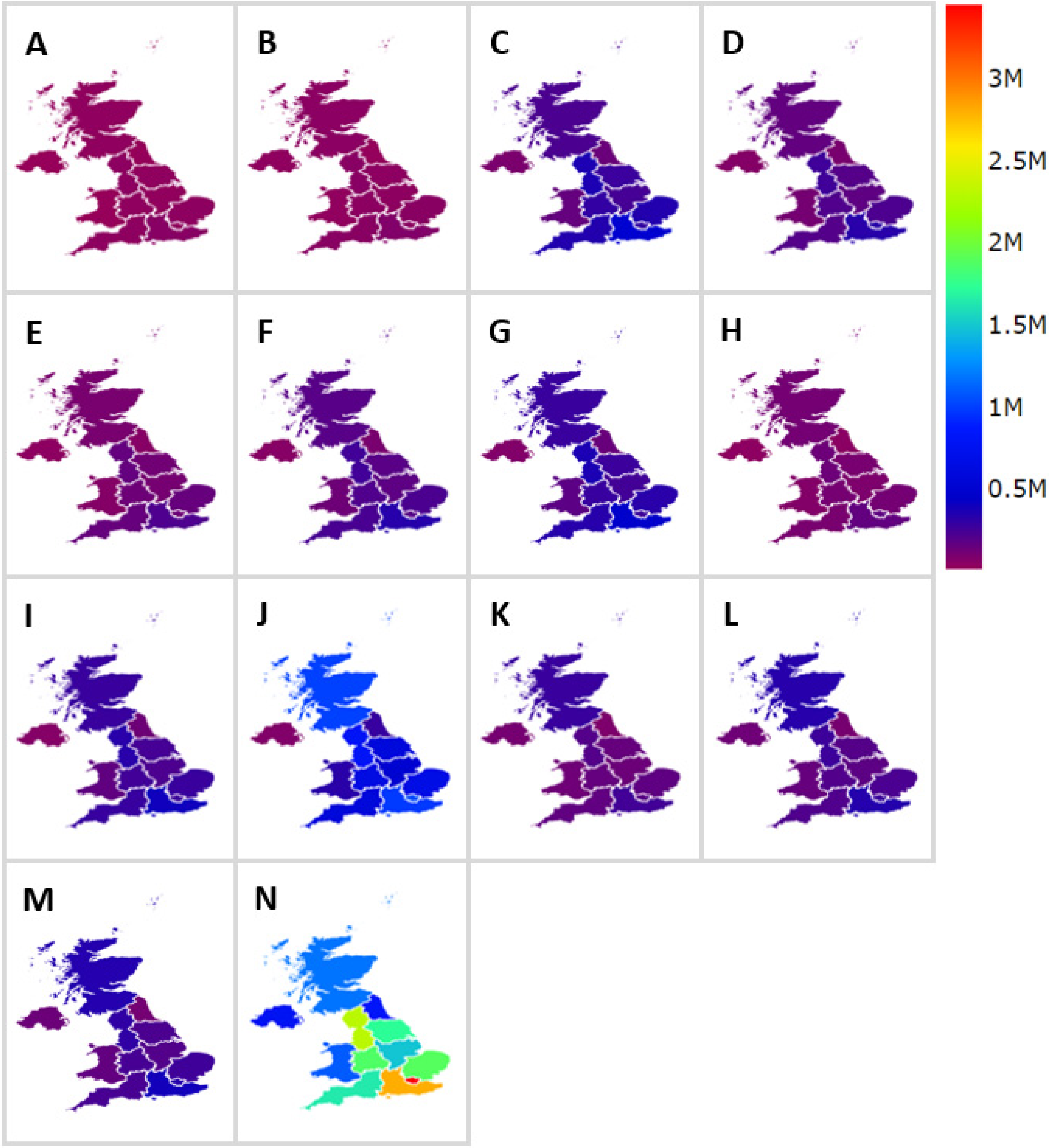

Figure S1. Spatial distribution of JCVI cohorts across Wales, Scotland, Northern Ireland, and the nine regions in England. A, Care home residence. B, Residential care workers. C, Individuals aged 80 and

over. D, Healthcare workers. E, Social care workers. F, Individuals aged 75-79. G, Individuals aged 70-74. H, Clinically extremely vulnerable under 70. I, individuals aged 65-69. J, At risk under 65. K, Individuals aged 60-64. L, Individuals aged 55-59. M, Individuals aged 50-54. N, Rest of adult population.

S1.2 Vaccine supply chain optimisation

S1.2.1 Weekly vaccine import planning

Vaccines are imported from manufacturing and fill-finish plants co-located at Puur, Belgium. **Figure S2** shows the weekly flow of BNT162b2 SARS-CoV-2 vaccine from plants to warehouses in London, Cardiff, Edinburgh, and Belfast.

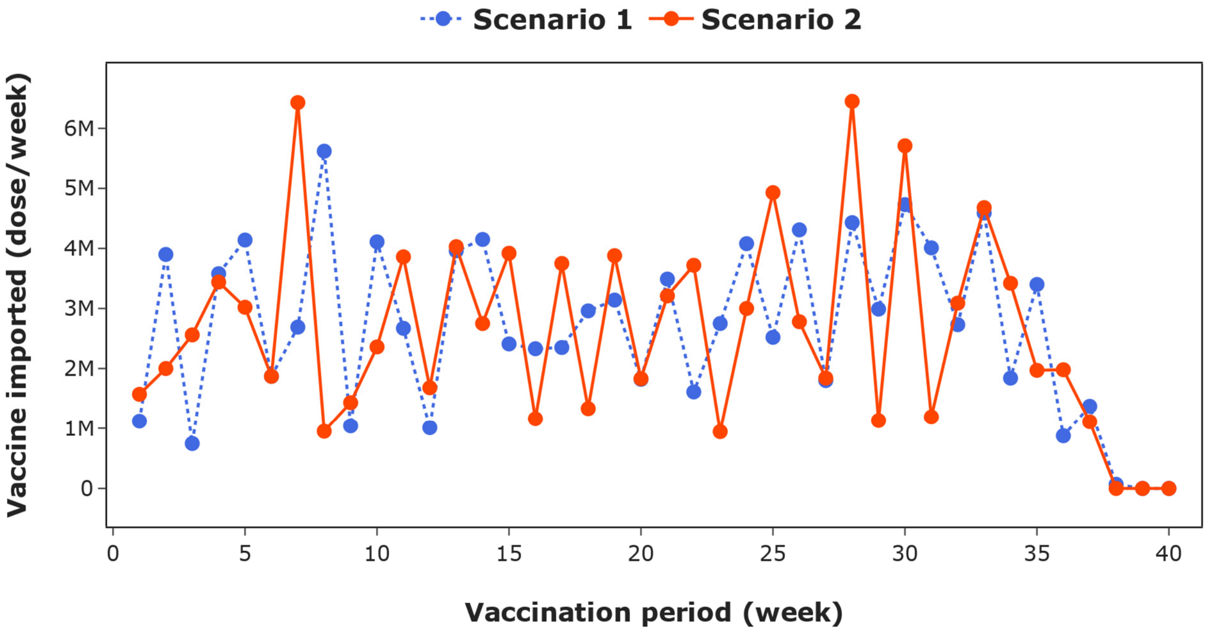

Figure S2. Vaccine import rate required to vaccinate adult population in the UK. The blue dotted curve denotes Scenario 1, whilst the red solid curve denotes Scenario 2.

The weekly import requirements range from 0 to 5.62 million dose week^{-1} and 0 to 6.45 million dose week^{-1} in Scenario 1 and Scenario 2 respectively. When the weekly import is zero, it means no vaccine is shipped from plants to warehouses. This can happen when vaccines stored in previous week(s) are used to satisfy demand of the prevailing week, consequently reducing the number of trips but increasing storage capacity. This trend is observed in Scenario 2. Both Scenarios 1 and 2 capitalises on the trade-off between transportation cost and storage cost to arrive at an optimal vaccine import rate. The results shown in **Figure S2** can be used to set production targets at both manufacturing and fill-finish plants, in addition to planning the necessary logistic to deliver the vaccines from plants to warehouses. Detailed discussions on logistics requirements are presented in Section S1.2.4.

S1.2.2 Quality control capacity planning

Maintaining high product quality is a mandatory requirement for processes producing biological products, including vaccines. In addition to the stringent quality control tests carried out during manufacturing and fill-finish, vaccines also undergo quality control checks at warehouses, especially if the vaccines arrive from abroad[1–3]. In this study, batches of BNT162b2 SARS-CoV-2 vaccine imported from factory in Puur, Belgium undergo quality control checks prior to distribution and delivery, which take about two weeks. **Figure S3** shows the weekly quality control capacity requirements at warehouses located in London, Edinburgh, Cardiff, and Belfast.

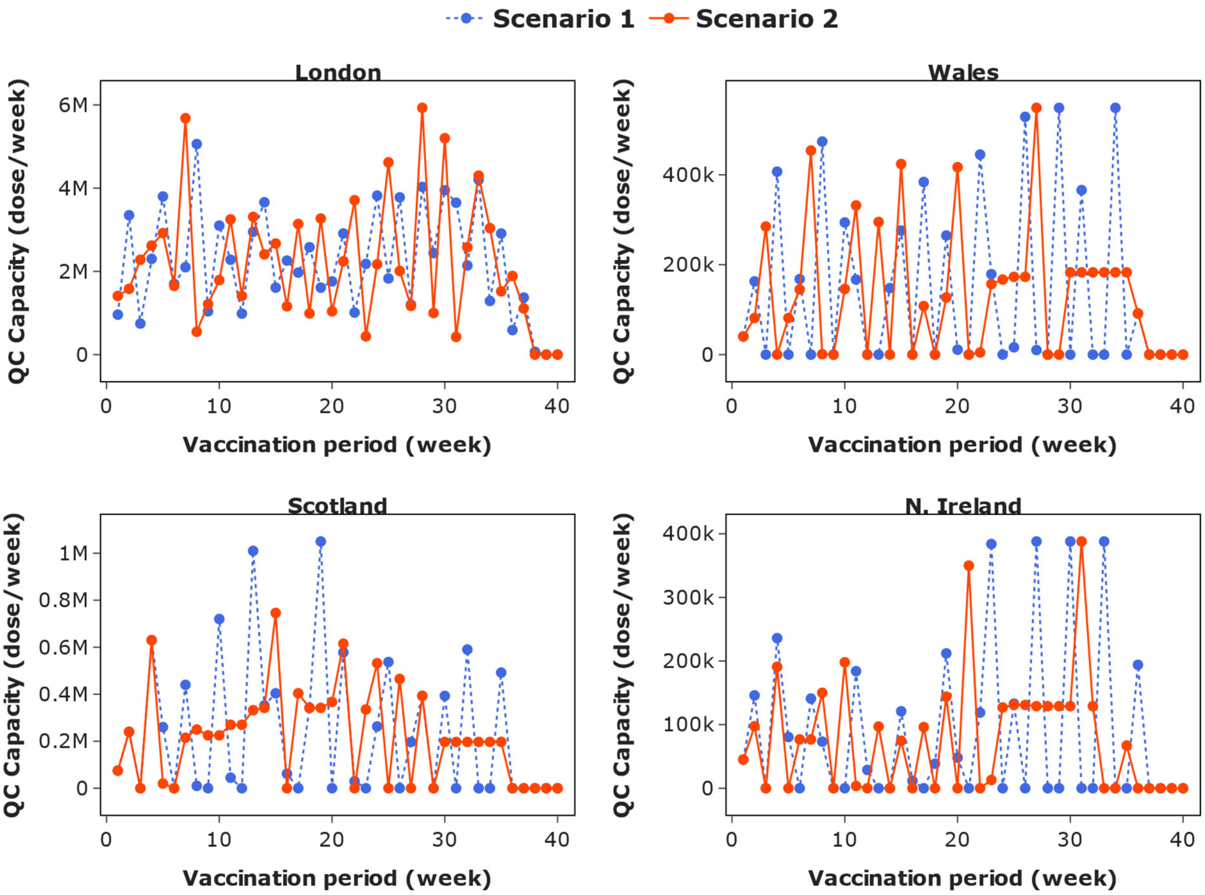

Figure S3. Quality control capacity required at warehouses across the UK. The blue dotted curve denotes Scenario 1, whilst the red solid curve denotes Scenario 2.

From the results, quality control capacity requirements (in million doses per week) vary on a weekly basis throughout the vaccination period. In Scenario 1, the capacity required in London, Wales, Scotland, and Northern Ireland corresponds to 5.060, 0.549, 1.050, and 0.388 respectively, whilst in Scenario 2, the capacities correspond to 5.930, 0.549, 0.746, and 0.388 respectively. This information is crucial when (i) building capacity for vaccine quality control test at warehouses, (ii) estimating the total cost of quality control, and (iii) estimating the human resources required.

S1.2.3 Handling vaccine thermal shippers during COVID-19 vaccination campaign

Due to the ultra-low temperature requirement for BNT162b2 SARS-CoV-2 vaccine, Pfizer and BioNTech have developed a specialised temperature control box called "thermal shipper" to enable the transport of their vaccine candidate from factory to vaccination centres, without compromising the stringent temperature requirements of -80°C. **Figure S4** shows the weekly flow of vaccine shippers from warehouses in London, Edinburgh, Cardiff, and Belfast to all vaccination centres located in each region.

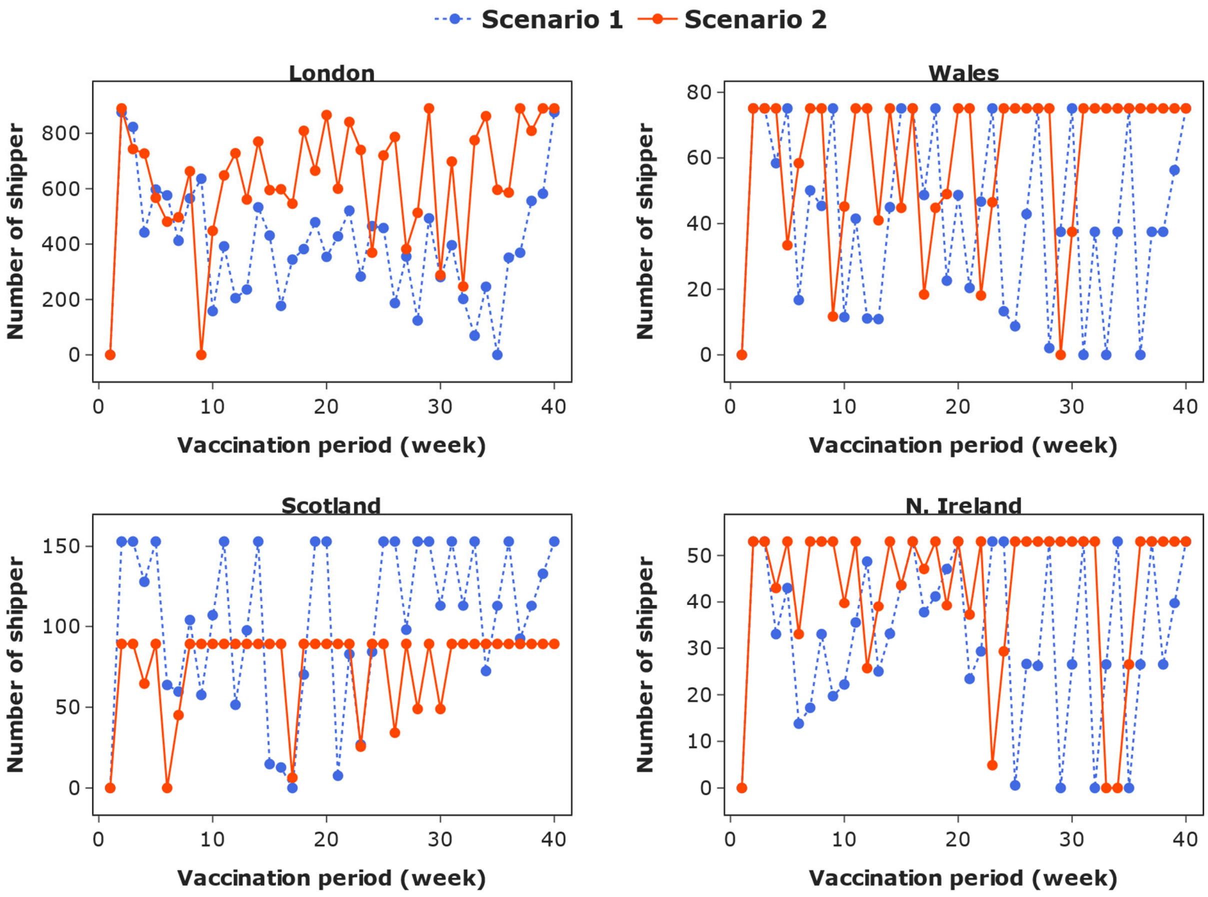

Figure S4. Weekly flow of vaccine shippers from warehouses to vaccination centres and back to warehouses. The blue dotted curve denotes Scenario 1, whilst the red solid curve denotes Scenario 2.

Across the UK, vaccination starts at week 3, therefore an initial quantity of vaccine shippers must be supplied to all warehouses in week 2. In Scenario 1, the optimal number of shippers that should be supplied to warehouses in London, Wales, Scotland, and Northern Ireland corresponds 876, 75, 153, and 53 respectively, while in scenario 2 the initial number of shippers corresponds 890, 75, 89, and 53 respectively. The total quantity of shippers required throughout the vaccination campaign is approximately 1,160 and 1,110 for scenarios 1 and 2 respectively. All empty shippers at clinics/vaccination centres are returned back to their corresponding warehouses for re-use. In this way, the total number of thermal shippers required can be reduced significantly.

S1.2.4 Distribution and transportation planning

Throughout the vaccination period, BNT162b2 SARS-CoV-2 vaccines are transported across various levels of the supply chain to satisfy demand at vaccination centres. In this work, three transport modes were used, namely, plane, refrigerated truck, and refrigerated van. To enable rapid shipment of BNT162b2 SARS-CoV-2 vaccine, only plane is used for shipment across international transport routes, i.e., routes from factory in Puur, Belgium to warehouses in London, Edinburgh, Cardiff, and Belfast. On the other hand, either truck or van can be used for domestic transport routes, i.e., routes from warehouses to regional stores, regional stores to clinics, and clinics to warehouses. **Figure S5** shows the total trips covered by transportation modes throughout the COVID-19 vaccination campaign in England, Scotland, Wales, and Northern Ireland.

The routes between warehouses to regional stores and regional stores to clinics are used for transporting full shippers, while only empty shippers are transported from clinics to warehouses. In both scenarios, only truck is used in routes between regional stores and clinics, and clinics to warehouses. Both truck and van are used in routes between warehouses and regional stores. **Figure S5** explain these observations and also shows the total trips covered by transport modes delivering BNT162b2 SARS-CoV-2 vaccines across various levels of the supply chain.

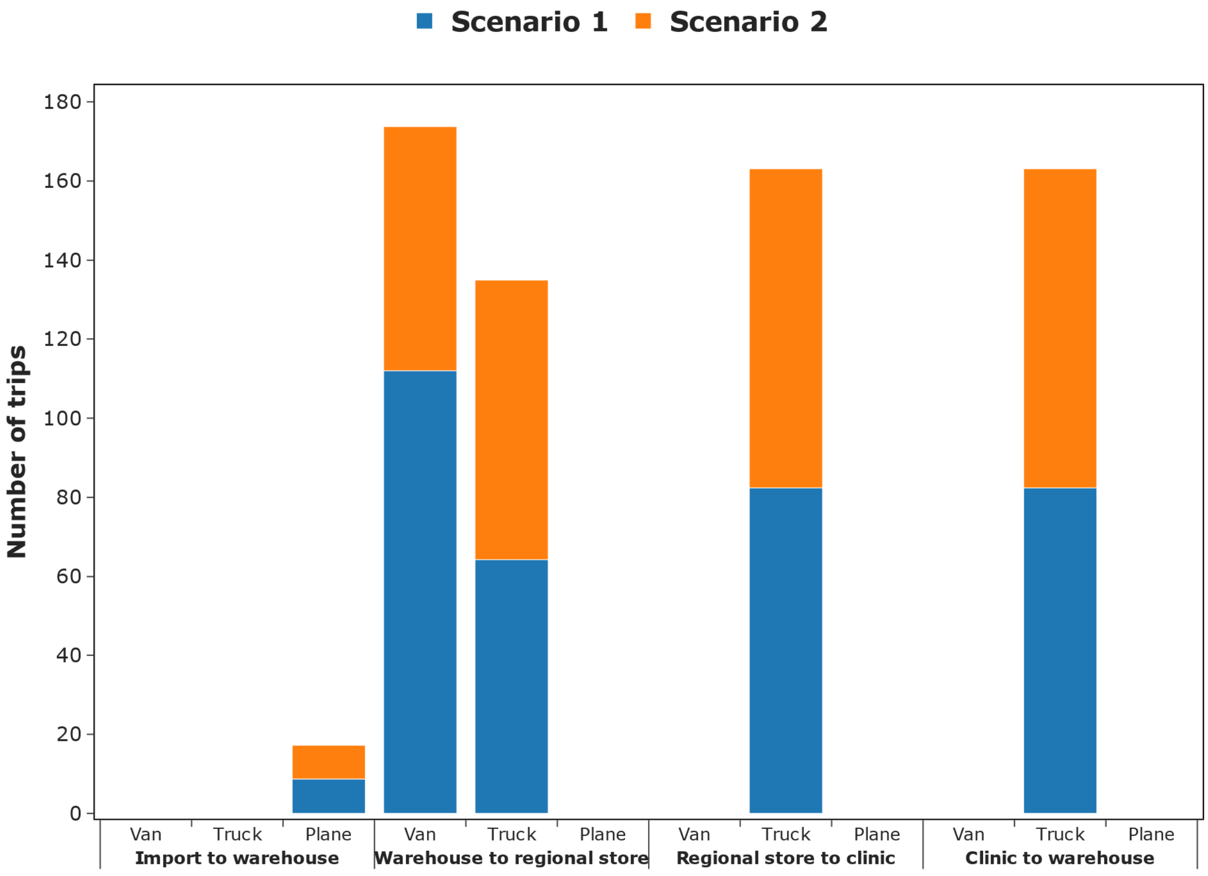

Figure S5. Total trips covered by transportation modes at various levels of the supply chain.

In routes between warehouses and regional stores, large numbers of trips are observed using refrigerated vans compared to trucks, particularly in Scenario 1. This can be attributed to the fact that the capacity of a refrigerated van (7,676L equivalent to 0.421 million doses per trip) used in this work is smaller than a refrigerated truck (47,424L equivalent to 2.601 million doses per trip), therefore for a given payload, a large number of trips will be required using vans. In addition, selection of transport mode when minimising logistics cost accounts for transportation cost, which is the product of unit transport cost and number of trips. Since unit transport cost per route is fixed, it is necessary to minimise the number of trips per route to achieve low transport cost, which is a major component of the total supply chain cost. Information presented in **Figure S5** can be used to facilitate transportation/distribution planning, for example, estimation of number and type of vehicle needed, quantity of fuel required, vehicle driver, and transport routes to follow during distribution.

Supplementary 2: Information related to vaccine characteristics and vaccine administration

Table S1. Characteristics of mRNA vaccine candidate considered in the work, i.e., BNT162b2 Sars-Cov-2 vaccine developed by Pfizer and BioNTech.

Item	Value/Description	Unit	Sources
Type	mRNA vaccine	[-]	
Abbreviation	BNT162b2	[-]	
Manufacturer	Pfizer-BioNTech	[-]	
Method of administration	Intramuscular	[-]	Ref[4]
Vaccine presentation	Liquid	[-]	Ref[4]
Storage temperature	-80	^{o}C	Ref[5,6]
Dose(s) per vial	5	dose/vial	Ref[4]
Primary packaging	195	vial/tray	Ref[7–9]
Secondary packaging	975	tray/shipper	Ref[7–9]
Vaccine cost	18.66	$/dose	Ref[10]
Cost of dry ice	2.89	$/kg	Ref[11]
Cost of thermal shipper	6,675	$/shipper	Ref[12]
Vaccination schedule	2 doses, administered three weeks apart	dose/patient	Ref[4]

The guide on vaccination programme in the UK is set out by the joint committee on vaccination and immunisation (JCVI). This includes the types of vaccines authorised by the medicine and healthcare regulatory agency (MHRA), vaccination regiment (i.e., interval between prime and booster doses), target population to be vaccinated, and the order in which the vaccination is conducted. Detailed information on how the JCVI develop this criterial can be found elsewhere[13]. Table S2 presents the JCVI cohorts used in this study, and their corresponding order/prioritisation. JCVI cohorts with the same priority are schedule for vaccination at the same time.

Table S2. Prioritisation according to groups recommended by the joint committee on vaccination and immunisation. Groups are classified as either Phase 1 or Phase 2.

Phase	JCVI Cohort	Priority Group
1	Care home residents	1
1	Residential Care workers	1
1	80+	2
1	Healthcare workers	2
1	Social care workers	2
1	75-79	3
1	70-74	4
1	Clinically extremely vulnerable (under 70)	4
1	65-69	5
1	At risk (under 65)	6
1	60-64	7
1	55-59	8
1	50-54	9
2	Rest of adult population	10 to 12

Supplementary 3: Details of supply chain modelling

The vaccine supply chain model developed in this work rely on the concept of echelon supply chain, where each echelon or supply level denotes a unique activity. The vaccine supply chain consists of five echelons: internal and external manufacturing and fill-finish plants, in-country warehouses, regional vaccine stores and administration points. The locations of manufacturing plants and administration points can be fixed or selected, whilst locations of fill-finish plant, imports, warehouses, regional stores are to be selected. Airfreights are used for international transport, while domestic transport are carried out using either refrigerated vans or refrigerated trucks. This section presents the vaccine supply chain model, starting with the description of indices, parameters, and variables used in the model, followed by the mathematical formulation.

Indices

m = manufacturing plants
i = import locations
f = fill-finish plants
w = warehouses
r = regional stores
c = administration points
v = vaccine
j = transport mode
t = time period

Parameter

$I1_m^{min}$ = Minimum capacity of manufacturing plant *m* (dose/week)
$I2_f^{min}$ = Minimum capacity of fill-finish plant *f* (dose/week)
$I3_w^{min}$ = Minimum capacity of warehouse *w* (dose/week)
$I4_r^{min}$ = Minimum capacity of regional store *r* (dose/week)
$I5_c^{min}$ = Minimum capacity of administration point *c* (dose/week)
$I1_m^{max}$ = Maximum capacity of manufacturing plant *m* (dose/week)
$I2_f^{max}$ = Maximum capacity of fill-finish plant *f* (dose/week)
$I3_w^{max}$ = Maximum capacity of warehouse *w* (dose/week)
$I4_r^{max}$ = Maximum capacity of regional store *r* (dose/week)
$I5_c^{max}$ = Maximum capacity of administration point *c* (dose/week)
D_{vct}^{N}= Vaccine *v* needed at administration point *c* at time period *t* (dose/week)
QC_{vwt}^{min} = Minimum vaccine *v* to undergo QC check at warehouse w at time period t (dose/week)
QC_{vwt}^{max} = Maximum vaccine *v* to undergo QC check at warehouse w at time period t (dose/week)
I_{vi}^{min} = Minimum quantity of vaccine *v* imported from location *i* (dose/week)
I_{vi}^{max} = Maximum quantity of vaccine *v* imported from location *i* (dose/week)
P_{vm}^{min} = Minimum production rate of vaccine *v* in manufacturing plant *m* (dose/week)
P_{vm}^{max} = Maximum production rate of vaccine *v* in manufacturing plant *m* (dose/week)
F_{vf}^{min} = Minimum fill-finish rate of vaccine *v* in fill-finish plant *f* (dose/week)
F_{vf}^{max} = Maximum fill-finish rate of vaccine *v* in fill-finish plant *f* (dose/week)
Q^{min} = Minimum vaccines to be transported between entities (dose)
Q^{max} = Maximum vaccines to be transported between entities (dose)

Q_{vj}^{max}=Maximum quantity of vaccine *v* to be shipped by transport mode *j*
$U1_{mft}$ = Unit transport cost from manufacturing *m* to fill-finish *f* at time period *t* (USD/dose/week)
$U2_{fwt}$ = Unit transport cost from fill-finish *f* to warehouse *w* at time period *t* (USD/dose/week)
$U3_{wrt}$ = Unit transport cost from warehouse *w* to regional store *r* at time period *t* (USD/dose/week)
$U4_{iwt}$ = Unit transport cost from import location *i* to warehouse *w* at time period *t* (USD/dose/week)
$U5_{rct}$ = Unit transport cost from regional store *r* to administration point *c* at time period *t* (USD/dose/week)
$U6_{cwt}$ = Unit transport cost from administration point *c* to warehouse *w* at time period *t* (USD/dose/week)
$C1_m$ = Unit capital cost of manufacturing plants (USD/facility/week)
$C2_f$ = Unit capital cost of fill-finish plants (USD/facility/week)
$C3_w$ = Unit capital cost of warehouses (USD/facility/week)
$C4_r$ = Unit capital cost of regional stores (USD/facility/week)
V_m^M = Unit operating cost of manufacturing plants (USD/dose/week)
V_f^F = Unit operating cost of fill-finish plants (USD/dose/week)
I_m^M = Unit inventory holding cost at manufacturing plants (USD/dose/week)
I_f^F = Unit inventory holding cost at fill-finish plants (USD/dose/week)
I_w^W = Unit inventory holding cost at warehouses (USD/dose/week)
I_r^R = Unit inventory holding cost at regional stores (USD/dose/week)
M= Scaling factor
DPS = Vaccine dose per shipper (dose/shipper)
UPS = Unit price of vaccine thermal shipper (USD/shipper)
DIS = Dry ice per shipper (kg)
UPD = Unit price dry ice (USD/kg)
UPV^a = Unit price of vaccine administration at clinics, pharmacies, hospitals, etc. (USD/patient)
UPV^b = Unit price of vaccine administration at care homes (USD/patient)
PPD = Unit price per vaccine dose (USD/dose)
UPQ = Price of QC checks (USD/dose)

Decision variables
$E1_m$ = one if manufacturing plant *m* is installed and zero otherwise
$E2_f$ = one if fill-finish plant *f* is installed and zero otherwise
$E3_w$ = one if warehouse *w* is installed and zero otherwise
$E4_i$ = one if import location *i* is selected and zero otherwise
$E5_r$ = one if regional store *r* is installed and zero otherwise
$E6_c$ = one if administration point *c* is installed and zero otherwise
$X1_{mf}$ = one if manufacturing plant *m* is linked to fill-finish plant *f*, and zero otherwise
$X2_{fw}$ = one if fill-finish plant *f* is linked to warehouse *w*, and zero otherwise
$X3_{wr}$ = one if warehouse *w* is linked to regional store *r*, and zero otherwise
$X4_{iw}$ = one if import location *i* is linked to warehouse *w*, and zero otherwise
$X5_{rc}$ = one if regional store *r* is linked to administration point *c*, and zero otherwise
$X6_{cw}$ = one if administration point *c* is linked to warehouse *w*, and zero otherwise
$Q1_{vmfjt}$ = Vaccine *v* shipped from manufacturing *m* to fill-finish *f* via mode *j* at time period *t* (dose)
$Q2_{vfwjt}$ = Vaccine *v* shipped from fill-finish plant *f* to warehouse *w* via mode *j* at time period *t* (dose)

$Q3_{vwrjt}$ = Vaccine *v* shipped from warehouse *w* to regional store *r* via mode *j* at time period *t* (dose)
$Q4_{viwjt}$ = Vaccine *v* shipped from import point *i* to warehouse *w* via mode *j* at time period *t* (dose)
$Q5_{vrcjt}$ = Vaccine *v* shipped from store *r* to administration point *c* via mode *j* at time period *t* (dose)
$Q6_{vwcjt}$ = Shipper for vaccine *v* shipped from administration point *c* to warehouse *w* via mode *j* at time period *t* (dose)
QC_{vwt}= Vaccine *v* undergoing quality control checks at warehouse *w* at time period *t* (dose)
P_{vmt} = Production rate of vaccine *v* in manufacturing plant m at time period *t* (dose/week)
D_{vct}^{S} = Vaccine *v* supplied to administration point *c* at time period *t* (dose/week)
M_{vit} = Vaccine *v* at import location *i* at time period *t* (dose/week)
$I1_{mt}$ = Inventory of vaccines in manufacturing plant *m* at time period *t* (dose/week)
$I2_{ft}$ = Inventory of vaccines in fill-to-finish plant *f* at time period *t* (dose/week)
$I3_{wt}$ = Inventory of vaccines in warehouse plant *w* at time period *t* (dose/week)
$I4_{rt}$ = Inventory of vaccines in regional store point *r* at time period *t* (dose/week)
$I5_{ct}$ = Inventory of vaccines in administration point *c* at time period *t* (dose/week)
NF_{wt}^{W}= Number of full shippers at warehouse w at time period *t* (shipper/week)
NE_{wt}^{W}= Number of empty shippers at warehouse *w* at time period *t* (shipper/week)
NF_{rt}^{R}= Number of full shippers at regional store *r* at time period *t* (shipper/week)
NF_{ct}^{C}= Number of full shippers at administration point *c* at time period *t* (shipper/week)
NE_{ct}^{C}= Number of empty shippers at administration point *c* at time period *t* (shipper/week)
W_{vct}^{C}= Expired vaccine v wasted at administration point *c* at time period *t* (dose/week)
$N1_{vmfjt}$=Number of trips from manufacturing plant *m* to fill-finish plant *f* via mode *j* at time period *t*
$N2_{vfwjt}$= Number of trips from fill-finish plant *f* to warehouse *w* via mode *j* at time period *t*
$N3_{vwrjt}$= Number of trips from warehouse *w* to regional store *r* via mode *j* at time period *t*
$N4_{viwjt}$= Number of trips from import location *i* to warehouse *w* via mode *j* at time period *t*
$N5_{vrcjt}$= Number of trips from regional store *r* to administration point *c* via mode *j* at time period *t*
$N6_{vcwjt}$= Number of trips from administration point *c* to warehouse *w* via mode *j* at time period *t*
D_{vct}^{A} = Actual demand of vaccine *v* at administration point *c* at time period *t* (dose/week)
B_{vct} = Backlog of vaccine *v* at administration point *c* at time period *t* (dose/week)
BL = Total backlog across all administration points and time periods (dose)
C_M = Capital cost of manufacturing plants (USD/week)
C_F = Capital cost of fill-finish plants (USD/week)
C_W = Capital cost of warehouses (USD/week)
C_R = Capital cost of regional stores (USD/week)
C_T = Total capital cost (USD/week)
V^T =Total operating cost (USD/week)
T_v^c = Total transport cost for vaccine *v* (USD/week)
V_{vct}^{A} = Availability of vaccine *v* at administration point *c* at time period *t* (%)
C_S = Cost of vaccine thermal shipper (USD)
C_D = Cost of dry ice (USD)
C_A^a = Cost of vaccine administration at clinics, pharmacies, hospitals, etc. (USD)
C_A^b = Cost of vaccine administration at care homes (USD)
C_V = Cost of vaccine procurement (USD)
C_Q = Cost of QC checks at warehouses (USD)

Model constraints: Network configuration

In the proposed mathematical formulation, binary variables *E* and *X* are used to install facilities and establish connections/routes between supply chain entities. Here *X* denotes the existence of routes between adjacent supply chain entities, for example, $X1_{mf}$ denotes the route between manufacturing plants and fill-finish plants. *E* denotes the existence of supply chain entities, for example $E1_m$, $E2_f$, $E3_w$, $E4_i$, $E5_r$, and $E6_c$ denote manufacturing plants, fill-finish plants, warehouses, import locations, regional stores, and administration points respectively. Equations 1 and 2 establish routes between manufacturing and fill-finish plants only if the manufacturing and fill-finish plants exist. The same concept applies to fill-finish plants to warehouses, import locations to warehouses, warehouses to regional stores, regional stores to administration points, and administration points to warehouses, see Equations 3 to 11.

$$X1_{mf} \leq E1_m \quad \forall\, mf \quad (1)$$
$$X1_{mf} \leq E2_f \quad \forall\, mf \quad (2)$$
$$X2_{fw} \leq E2_f \quad \forall\, fw \quad (3)$$
$$X2_{fw} \leq E3_w \quad \forall\, fw \quad (4)$$
$$X4_{iw} \leq E3_w \quad \forall\, iw \quad (5)$$
$$X4_{iw} \leq E4_i \quad \forall\, iw \quad (6)$$
$$X3_{wr} \leq E3_w \quad \forall\, wr \quad (7)$$
$$X3_{wr} \leq E5_r \quad \forall\, wr \quad (8)$$
$$X5_{rc} \leq E5_r \quad \forall\, rc \quad (9)$$
$$X5_{rc} \leq E6_c \quad \forall\, rc \quad (9)$$
$$X6_{cw} \leq E3_w \quad \forall\, cw \quad (10)$$
$$X6_{cw} \leq E6_c \quad \forall\, cw \quad (11)$$

Vaccines shipped between facilities

Vaccines are shipped between supply chain entities using three types of transportation modes: refrigerated van, refrigerated truck, and airfreight. The index *j* in Equations 12 to 17 represents the set of transportation modes. In Equation 12, when a route is established between manufacturing plant *m* and fill-finish plant *f*, vaccines are allowed to be shipped using a specific transport mode. $Q1_{vmfjt}$ denotes the quantity of vaccine *v* shipped from manufacturing plant *m* to fill-finish plant *f* using transportation mode *j* at time period *t*. Equations 13 to 17 define the shipment of vaccines/shippers between fill-finish plants and warehouses, warehouses and regional stores, import location and warehouses, regional stores and administration points, administration points and warehouses respectively.

$$Q1_{vmfjt} \leq X1_{mf} \cdot M \quad \forall\, vmfjt \quad (12)$$
$$Q2_{vfwjt} \leq X2_{fw} \cdot M \quad \forall\, vfwjt \quad (13)$$
$$Q3_{vwrjt} \leq X3_{wr} \cdot M \quad \forall\, vwrjt \quad (14)$$
$$Q4_{viwjt} \leq X4_{iw} \cdot M \quad \forall\, viwjt \quad (15)$$
$$Q5_{vrcjt} \leq X5_{rc} \cdot M \quad \forall\, vrcjt \quad (16)$$
$$Q6_{vcwjt} \leq X6_{cw} \cdot M \quad \forall\, vcwjt \quad (17)$$

Model constraints: Material balance

The inlet and outlet flow of vaccines for each supply chain entity can be estimated by conducting a material balance over the entity. Here, entity refers to manufacturing plants, fill-finish plants, warehouses, regional stores, and administration points. For example, in Equation 18, $I1_{mt}$ denotes the inventory of vaccines in manufacturing plant *m* at time period *t*, which is calculated as the quantity of vaccine *v* produced at manufacturing plant *m* ($F1_{vmt} = P_{vmt}$) at time *t* minus the quantity of vaccine leaving the plant at time *t* ($F2_{vmt} = \sum_{mj} Q1_{vmfjt}$) plus the inventory of vaccines in the previous time period ($I1_{mt-1}$). Similarly, Equations 19 to 23 are used to estimate the inventory of vaccines in fill-finish plants, warehouses, regional stores, and administration points, where $F3_{vft} = \sum_{mj} Q1_{vmfjt}$; $F4_{vft} = \sum_{wj} Q2_{vfwjt}$; $F5_{vwt} = \sum_{fj} Q2_{vfwjt} + \sum_{ij} Q4_{viwjt}$; $F6_{vwt} = \sum_{rj} Q3_{vwrjt}$; $F7_{vrt} = \sum_{wj} Q3_{vwrjt}$; $F8_{vrt} = \sum_{cj} Q5_{vrcjt}$; $F9_{vct} = \sum_{rj} Q5_{vrcjt}$; $F10_{vct} = D^S_{vct}$; and $M_{vit} = \sum_{wj} Q4_{viwjt}$.

In warehouses, vaccines are expected to undergo quality control (QC) checks, which can take up to two weeks. To account for the delay due to QC, the inventory of vaccines at warehouse is partitioned into $I3in_{wt}$ and $I3out_{wt}$, denoting vaccine inventory before and after QC respectively. The inventory of vaccines before QC is calculated by subtracting the quantity of vaccines leaving for QC checks from the sum of vaccines arriving from fill-finish plants, oversees imports, and vaccine inventory at previous time period. The inventory of vaccines after QC is calculated by subtracting the quantity of vaccines leaving warehouses from vaccines that have undergoes QC checks and vaccine inventory at previous time period. The negative two subscript indicates that only vaccines that stays for two weeks are allowed to leave warehouses. Here, each time period is equivalent to one week.

$$I1_{mt} = \sum_v F1_{vmt} - \sum_v F2_{vmt} + I1_{mt-1} \qquad \forall\, mt \qquad (18)$$
$$I2_{ft} = \sum_v F3_{vft} - \sum_v F4_{vft} + I2_{ft-1} \qquad \forall\, ft \qquad (19)$$
$$I3in_{wt} = \sum_v F5_{vwt} - \sum_v QC_{vwt} + I3in_{wt-1} \qquad \forall\, wt \qquad (20)$$
$$I3out_{wt} = \sum_v QC_{vwt-2} - \sum_v F6_{vwt} + I3out_{wt-1} \qquad \forall\, wt \qquad (21)$$
$$I4_{rt} = \sum_v F7_{vrt} - \sum_v F8_{vrt} + I4_{rt-1} \qquad \forall\, rt \qquad (22)$$
$$I5_{ct} = \sum_v F9_{vct} - \sum_v F10_{vct} - \sum_v W^C_{vct} + I5_{ct-1} \qquad \forall\, ct \qquad (23)$$

Tracking vaccine shippers: warehouses–regional store–clinics–warehouses

Similar to vaccines, the flow of thermal shippers between supply chain entities are calculated using Equations 24 to 28. Equations 24, 26, and 27 are used to calculate the number of full shippers in warehouses, regional stores, and administration points, whereas Equations 25 and 28 calculate the number of empty shippers. The number of full shippers in warehouses, regional stores, and administration points is equal to shippers arriving plus shippers from pervious time period minus shippers leaving the facilities. Likewise, the number of empty shippers equal to empty shippers arriving plus empty shippers from previous time period minus empty shippers loaded with vaccines ready to be shipped from warehouses.

$$NF^W_{wt} = \frac{\sum_v QC_{vwt-2}}{DPS} - \frac{\sum_v F6_{vwt}}{DPS} + \frac{\sum_{vij} Q4_{viwjt}}{DPS} + NF^W_{wt-1} \qquad \forall\, wt \qquad (24)$$
$$NE^W_{wt} = \frac{\sum_{vcj} Q6_{vcwjt}}{DPS} - \frac{\sum_v F5_{vwt}}{DPS} - \frac{\sum_{vij} Q4_{viwjt}}{DPS} + NE^W_{wt-1} \qquad \forall\, wt \qquad (25)$$
$$NF^R_{rt} = \frac{\sum_v F7_{vrt}}{DPS} - \frac{\sum_v F8_{vrt}}{DPS} + NF^R_{rt-1} \qquad \forall\, rt \qquad (26)$$

$$NF_{ct}^{C} = \frac{\sum_v F9_{vct}}{DPS} - \frac{\sum_v F10_{vct}}{DPS} - \frac{\sum_v W_{vct}^{C}}{DPS} + NF_{ct-1}^{C} \qquad \forall\, ct \qquad (27)$$

$$NE_{ct}^{C} = \frac{\sum_v F10_{vct}}{DPS} - \frac{\sum_{vwj} Q6_{vcwjt}}{DPS} + \frac{\sum_v W_{vct}^{C}}{DPS} + NE_{ct-1}^{C} \qquad \forall\, ct \qquad (28)$$

Shelf life of vaccines at administration points

COVID-19 vaccine developed by Pfizer and BioNTech have a shelf life of one week when stored inside a thermal shipper, and requires a sufficient quantity of dry ice to keep the temperature at -80°C. Equation 29 ensures that vaccines stay at administration points for one week only. Any unused vaccines are discarded as waste after one week.

$$NF_{ct}^{C} \leq \frac{\sum_{vt}^{t+1} D_{vct}^{S}}{DPS} \qquad \forall\, ct \qquad (29)$$

Safety stock, maximum inventory, and bounds on QC checks, production rate, and fill-finish rate

Safety stocks are used to compliment vaccine supply, especially when there is a supply shortage during a vaccination campaign. Equations 30 to 34 define the minimum inventory of vaccine at each facility within the supply chain.

$$I1_{mt} \geq I1_{m}^{min} \cdot E1_{m} \qquad \forall\, mt \qquad (30)$$

$$I2_{ft} \geq I2_{f}^{min} \cdot E2_{f} \qquad \forall\, ft \qquad (31)$$

$$I3in_{wt} + I3out_{wt} \geq I3_{w}^{min} \cdot E3_{w} \qquad \forall\, wt \qquad (32)$$

$$I4_{rt} \geq I4_{r}^{min} \cdot E5_{r} \qquad \forall\, rt \qquad (33)$$

$$I5_{ct} \geq I5_{c}^{min} \qquad \forall\, ct \qquad (34)$$

Similarly, Equations 35 to 39 defines the maximum inventory of vaccines that can be stored at each supply chain entity.

$$I1_{mt} \leq I1_{m}^{max} \cdot E1_{m} \qquad \forall\, mt \qquad (35)$$

$$I2_{ft} \leq I2_{f}^{max} \cdot E2_{f} \qquad \forall\, ft \qquad (36)$$

$$I3in_{wt} + I3out_{wt} \leq I3_{w}^{max} \cdot E3_{w} \qquad \forall\, wt \qquad (37)$$

$$I4_{rt} \leq I4_{r}^{max} \cdot E5_{r} \qquad \forall\, rt \qquad (38)$$

$$I5_{ct} \leq I5_{c}^{max} \qquad \forall\, ct \qquad (39)$$

Equations 40 to 47 defines the minimum and maximum QC capacity at warehouses, quantity of vaccines imported from oversees, production rate at manufacturing plants, and fill-finish rate at fill-finish plants, respectively.

$$\sum_v QC_{vwt} \geq QC_{vw}^{min} \qquad \forall\, vwt \qquad (40)$$

$$\sum_v QC_{vwt} \leq QC_{vw}^{max} \qquad \forall\, vwt \qquad (41)$$

$$M_{vit} \geq I_{vi}^{min} \qquad \forall\, vit \qquad (42)$$

$$M_{vit} \leq I_{vi}^{max} \qquad \forall\, vit \qquad (43)$$

$$P_{vmt} \geq P_{vm}^{min} \qquad \forall\, vmt \qquad (44)$$

$$P_{vmt} \leq P_{vm}^{max} \qquad \forall\, vmt \qquad (45)$$

$$F3_{vft} \geq F_{vf}^{min} \qquad \forall\, vft \qquad (46)$$

$$F3_{vft} \leq F_{vf}^{max} \qquad \forall\, vft \qquad (47)$$

Minimum and maximum vaccine transported

Equations 48 to 53 define the minimum and maximum possible flow of vaccines between supply chain entities. Q^{min} is fixed at zero, whereas Q^{max} is fixed 10^{10}.

$$Q^{min} \leq Q1_{vmfjt} \leq Q^{max} \qquad \forall\ vmfjt \qquad (48)$$
$$Q^{min} \leq Q2_{vfwjt} \leq Q^{max} \qquad \forall\ vfwjt \qquad (49)$$
$$Q^{min} \leq Q3_{vwrjt} \leq Q^{max} \qquad \forall\ vwrjt \qquad (50)$$
$$Q^{min} \leq Q4_{viwjt} \leq Q^{max} \qquad \forall\ viwjt \qquad (51)$$
$$Q^{min} \leq Q5_{vrcjt} \leq Q^{max} \qquad \forall\ vrcjt \qquad (52)$$
$$Q^{min} \leq Q6_{vcwjt} \leq Q^{max} \qquad \forall\ vrcjt \qquad (53)$$

Number of trips

Equation 54 to 59 calculate the number of trips between entities. Here $N1_{vmfjt}$, $N2_{vfwjt}$, $N3_{vwrjt}$, $N4_{viwjt}$, $N5_{vrcjt}$, and $N6_{vcwjt}$ denote the number of trips between manufacturing plant and fill-finish plant, fill-finish plant and warehouses, warehouses to regional stores, imports to warehouses, regional stores to clinic, and clinics to warehouses.

$$N1_{vmfjt} = \frac{Q1_{vmfjt}}{Q_{vj}^{max}} \cdot 2 \qquad \forall\ vmfjt \qquad (54)$$
$$N2_{vfwjt} = \frac{Q2_{vfwjt}}{Q_{vj}^{max}} \cdot 2 \qquad \forall\ vfwjt \qquad (55)$$
$$N3_{vwrjt} = \frac{Q3_{vwrjt}}{Q_{vj}^{max}} \cdot 2 \qquad \forall\ vwrjt \qquad (56)$$
$$N4_{viwjt} = \frac{Q4_{viwjt}}{Q_{vj}^{max}} \cdot 2 \qquad \forall\ viwjt \qquad (57)$$
$$N5_{vrcjt} = \frac{Q5_{vrcjt}}{Q_{vj}^{max}} \cdot 2 \qquad \forall\ vrcjt \qquad (58)$$
$$N6_{vcwjt} = \frac{Q6_{vcwjt}}{Q_{vj}^{max}} \cdot 2 \qquad \forall\ vcwjt \qquad (59)$$

Non-negativity constraints

In the supply chain model, all variables have non-negative values and this condition is enforced by Equation 60.

$$All\ variables \geq 0 \qquad (60)$$

Objective function:

There are many criteria that can be used to assess the performance of vaccine supply chains; however, this work considers three important and relevant performance measures: backlog, logistics cost and/or total cost, and vaccine availability.

Backlog

Backlog is the sum of un-fulfilled vaccinations which occurs as a result of shortage in vaccine supply. Equation 63 calculates the total backlog, i.e., the sum of un-fulfilled vaccination over the entire vaccination period. Backlog at vaccine administration points can be calculated by subtracting actual vaccine demand from vaccine supplied (see Equation 62), where actual demand is the sum of

vaccine needed (defined by the number of appointments registered at administration points) and backlog from previous time period (see Equation 61).

$$D_{vct}^{A} = D_{vct}^{N} + B_{vct-1} \tag{61}$$
$$B_{vct} = D_{vct}^{A} - D_{vct}^{S} \tag{62}$$
$$BL = \sum_{vct} B_{vct} \tag{63}$$

Capital investment cost

Logistics cost is the sum of annualised capital cost, operating cost, and transportation cost. Annualised capital cost is the sum of installation cost of supply chain entities, including manufacturing plants, fill-finish plants, warehouses, and regional stores. Equation 64 calculates the total annualised capital cost.

$$C_T = \sum_m E1_m \cdot C1_m + \sum_f E2_f \cdot C2_f + \sum_w E3_w \cdot C3_w + \sum_r E4_r \cdot C4_r \tag{64}$$

Operating cost

The operating cost is divided into process operating cost and inventory holding cost. Process operating cost relates to manufacturing plants and fill-finish plants, and is calculated as vaccine production rate multiply by unit production cost and fill-finish rate multiply by unit fill-finish cost. On the other hand, inventory holding cost is define as the prevailing inventory multiply by unit inventory holding cost. Equation 65 calculates the total operating cost.

$$V^T = \sum_{vmt} P_{vmt} \cdot V_m^M + \sum_{vft} F3_{vft} \cdot V_f^F + \sum_{mt} I1_{mt} \cdot I_m^M + \sum_{ft} I2_{ft} \cdot I_f^F + \sum_{wt} (I3in_{wt} + I3out_{wt}) \cdot I_w^W + \sum_{rt} I4_{rt} \cdot I_r^R \tag{65}$$

Transport cost

Equation 66 calculates the total transportation cost between supply chain entities, which is define as the unit transportation cost for a specific transport mode multiply by the number of trips covered at a given time period. The unit transportation cost accounts for travel distance, driver wages, fuel and vehicle maintenance cost, and annualised capital cost.

$$T_v^c = \sum_{mfjt} U1_{mfj} \cdot N1_{vmfjt} + \sum_{fwjt} U2_{fwj} \cdot N2_{vfwjt} + \sum_{wrjt} U3_{wrj} \cdot N3_{vwrjt} + \sum_{iwjt} U4_{iwj} \cdot N4_{viwjt} + \sum_{rcjt} U5_{rcj} \cdot N5_{vrcjt} + \sum_{cwjt} U6_{cwj} \cdot N6_{vcwjt} \tag{66}$$

Vaccine availability at administration points

Equation 67 estimates vaccine availability at a specific administration point, defined as the quantity of vaccine supply to the administration point at a given time period divided by vaccine needed (defined by the number of appointments registered at administration points) at the same time period.

$$V_{vct}^{A} = \frac{D_{vct}^{S}}{D_{vct}^{N}} \cdot 100 \tag{67}$$

Cost of supply chain components

Equations 68 to 73 estimate the cost of supply chain components such as thermal shippers, dry ice, wages for administering vaccines (at care home and other vaccine administration points such as pharmacy, hospitals, GP surgeries, Clinics, etc.), vaccine procurement, and quality control checks. The cost of vaccine shippers can be estimated by multiplying the number of shippers supplied at the start of the vaccination campaign by the unit price of shipper (see Equation 68). Here, it is assumed that no shipper is damage and/or replace throughout the vaccination campaign. The cost of dry ice can be calculated as the total dry ice required multiply by the unit price of dry ice (see Equation 69); each full shipper leaving warehouses is loaded with sufficient dry ice to maintain the sub-zero storage temperature of -80°C. Equation 70 calculates the cost of administering vaccine, while Equation 71 accounts for the extra cost when vaccines are administered at care homes. The total cost of vaccine procured is define as the total doses supplied multiply by unit price per dose (see Equation 72), and lastly the cost of quality control checks is defined as the total vaccines that undergo quality control checks at warehouse multiply by the unit price of QC (see Equation 63).

$$C_S = \sum_{wt=2} NE^{W}_{wt} \cdot UPS \quad (68)$$
$$C_D = \sum_{vwrjt} \frac{Q3_{vwrjt}}{DPS} \cdot DIS \cdot UPD \quad (69)$$
$$C^{a}_{A} = \sum_{vct} D^{S}_{vct} \cdot UPV^{a} \quad (70)$$
$$C^{b}_{A} = \sum_{vct=3} D^{S}_{vct} \cdot UPV^{b} \cdot 2 \quad (71)$$
$$C_V = \sum_{vct} D^{S}_{vct} \cdot PPD \quad (72)$$
$$C_Q = \sum_{vwt} F5_{vwt} \cdot UPQ \quad (73)$$

Supplementary 4: Modelling vaccine administration and demand stratification

Prior to the COVID-19 vaccination campaign, the number of healthcare workers (HCWs) required as well as the timeframe for the completion of the vaccination campaign needs to be determined in order to enable an efficient planning and delivery of the vaccination exercise. Equation 74 estimates the number of HCWs that would be needed at each vaccine administration point on weekly basis, while Equation 75 determines the approximate timescale for administering prime and booster jabs to each cohort. This information is vital as it can be used to (i) develop a vaccination schedule, which provides time slot for appointment of target individuals/cohorts on daily or weekly basis, (ii) develop a daily/weekly duty roster for HCWs and provides an estimate of the total number of HCWs that should be recruited for the vaccination exercise.

$$HCW_{ct} = \frac{TP_{ct}}{WL} \quad (74)$$

In Equation 74, HCW_{ct} denotes the daily or weekly, t, number of HCWs needed at vaccination point *c* to administer vaccines to target patients, TP_{ct} denotes target population arriving at vaccine administration point *c* at time period *t*, and WL denotes workload. Workload is defined as administration rate divided by staff available working hours.

$$VT_a = \frac{TP_a}{VR} \quad (75)$$

In Equation 75, VT_a denotes the vaccination timeframe for target population/cohorts *a* (day or week), TP_a denotes the total target population/cohorts *a* (patient), VR is the vaccine administration rate target (patients/day or patients/week).

The demand stratification studies define the total target population/cohorts, TP_a, as well as the target population arriving at vaccine administration point *c* at time period t, TP_{ct}. For the UK studies, the cohorts recommended by the joint committee on vaccination and immunisation[13] is presented in **Table S2**, while individuals belonging to each cohort in all regions within the UK are collected from the UK's Office for National Statistics[14].

References

1. Biologicals, O. Regulation of vaccines : building on existing drug DEPARTMENT OF VACCINES AND. (1999).
2. Mao, Q. *et al.* COVID-19 vaccines: progress and understanding on quality control and evaluation. *Signal Transduct. Target. Ther.* **6**, (2021).
3. STRENGTHENING REGULATORY SYSTEMS Import testing. https://www.ifpma.org/subtopics/import-testing/ (2021).
4. REG 174 INFORMATION FOR UK HEALTHCARE PROFESSIONALS. https://assets.publishing.service.gov.uk/government/uploads/system/uploads/attachment_data/file/987586/Temporary_Authorisation_HCP_Information_BNT162_10_0.pdf (2021).
5. Thermal, R. & Containers, S. Guidance for Receiving and Handling the Pfizer-BioNTech COVID-19 mRNA Vaccine (including dry ice procedures) Table of Contents. 1–12 (2021).
6. PFIZER AND BIONTECH SUBMIT COVID-19 VACCINE STABILITY DATA AT STANDARD FREEZER TEMPERATURE TO THE U.S. FDA. https://www.pfizer.com/news/press-release/press-release-detail/pfizer-and-biontech-submit-covid-19-vaccine-stability-data (2021).
7. Since January 2020 Elsevier has created a COVID-19 resource centre with free information in English and Mandarin on the novel coronavirus COVID- 19 . The COVID-19 resource centre is hosted on Elsevier Connect , the company ' s public news and information . (2020).
8. Middleton, E. L. FACT SHEET FOR HEALTHCARE PROVIDERS ADMINISTERING VACCINE. **2019**, 1–37 (2019).
9. England, P. H. COVID-19 vaccination programme Information for healthcare practitioners About Public Health England. 1–40 (2020).
10. Wouters, O. J. *et al.* Challenges in ensuring global access to COVID-19 vaccines: production, affordability, allocation, and deployment. *Lancet* **397**, 1023–1034 (2021).
11. 10KG Dry ICE Block HIGH Density Solid CO2. https://www.amazon.co.uk/10KG-DRY-ICE-Block-Density/dp/B079LLT9HR (2021).
12. Sofrigam offers solutions to Covid-19 vaccines cold chain transport challenges. https://www.pharmaceutical-networking.com/sofrigam-covid-19-vaccine-cold-chain-

transport-solutions/ (2021).

13. Joint Committee on Vaccination and Immunisation: advice on priority groups for COVID-19 vaccination, 30 December 2020. https://www.gov.uk/government/publications/priority-groups-for-coronavirus-covid-19-vaccination-advice-from-the-jcvi-30-december-2020/joint-committee-on-vaccination-and-immunisation-advice-on-priority-groups-for-covid-19-vaccination-30-december-2020 (2020).

14. office for national statistics. https://www.ons.gov.uk/.
